# Multilevel Analysis of Ground Beetle Responses to Forest Management: Integrating Species Composition, Morphological Traits and Developmental Instability

**DOI:** 10.1002/ece3.70793

**Published:** 2025-01-21

**Authors:** Dominik Stočes, Jan Šipoš

**Affiliations:** ^1^ Department of Zoology, Fisheries, Hydrobiology and Apiculture, Faculty of Agronomy Mendel University in Brno Brno Czech Republic

**Keywords:** Bayesian statistics, fluctuating asymmetry, forest management practises, functional traits, wing morphology

## Abstract

This study evaluates the response of ground beetle (Coleoptera: Carabidae) assemblage to forest management practices by integrating species composition, body traits, wing morphology and developmental instability. Traditional approaches that rely on averaged identity‐based descriptors often overlook phenotypic plasticity and functional trait variability, potentially masking species‐specific responses to environmental changes. To address this, we applied a three‐layered analytical approach to address this gap, utilising ground beetle occurrence and morphological trait data from Podyjí National Park, Czech Republic. The first layer assessed assemblage composition with ecological and dietary preferences across control, ecotone and clearing treatments using multivariate techniques. Building on species‐level knowledge, the second layer analysed the interaction between coarse traits, such as wing morphology and fine‐scale body traits, including body size (proxied by elytron length), head width and last abdominal sternite, to assess their relationship with the different treatments. These interactions were explored as intraspecific wing plasticity can affect functional interpretations. The third layer focused on fluctuating asymmetry as an intraindividual indicator of developmental instability, examining how ground beetles respond to environmental stressors. Our findings revealed: (i) no significant impact of habitat treatments on the presence of specialist species in the assemblage analysis; (ii) analysis of morphological traits highlights the combined influence of a coarse trait, such as wing morphology, and a fine trait, such as head width, which together contribute to the partitioning of assemblages and help distinguish differences in habitat use; and (iii) FA analysis revealed a significant positive association between the second antennal segment of specialist species and litter while displaying a negative association with Collembola. This multilevel analytical framework not only confirms ecological findings but also advances our approach to habitat and species analysis, offering deeper insights into ecosystem dynamics.

## Graphical abstract

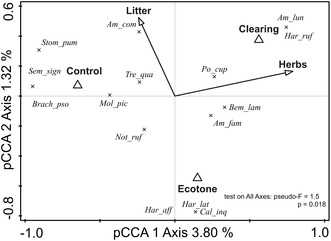

## 1 Introduction

In temperate forest ecosystems, human interventions such as forest thinning, regeneration felling and timber harvesting play a key role in shaping habitat structure and biodiversity (Thom and Seidl 2016; Oettel and Lapin 2021). Among these, timber harvesting of mature stands, particularly clear cutting, alters ecosystems by reducing tree density and modifying microclimatic conditions such as light penetration, soil moisture and temperature (Grevé et al. 2018). These environmental changes cascade through the ecosystem, affecting vegetation and invertebrate assemblages, particularly species that rely on stable forest conditions for survival and reproduction (Tigreros et al. 2023).

Clear cutting promotes the growth of early successional species and creates ecotones that support unique species assemblages (Kuuluvainen 2002; Harper et al. 2005; Pinksen et al. 2021). This increased habitat heterogeneity profoundly influences ground beetles (Carabidae), which are highly sensitive to environmental changes and serve as bioindicators of habitat quality (Lövei and Sunderland 1996; Rischen et al. 2022).

Ground beetles are frequently used to assess the impacts of forestry management practices on biodiversity due to their sensitivity to environmental shifts (Lange et al. 2014; Baptista et al. 2024). Studies have demonstrated that large‐scale forestry practices, such as clear cutting, significantly modify ground beetle assemblages by altering microhabitats and resource availability (Ribera et al. 2001; Weller and Ganzhorn 2004; Moretti et al. 2017). While these studies provide valuable insights into shifts in species assemblages (Moretti and Legg 2009; Pedley and Dolman 2014; Fournier et al. 2015; Baptista et al. 2024), they often rely on community‐level metrics that average species‐specific data. Although this approach captures broad ecological patterns, it may obscure key interactions at the species level and within individuals, particularly intraindividual variations in response to environmental stressors (Ribera et al. 2001; Bolnick et al. 2011; Elek, Růžičková, and Ódor 2021; Yang, Deng, and Lin 2023).

To address these limitations, it is essential to explore the interactions between coarse traits, such as phenotypic plasticity, and fine‐scale functional traits, such as body measurements, which provide a more detailed understanding of the ecological roles species play in their environments (Barton et al. 2011; Bolnick et al. 2011; Brousseau, Gravel, and Handa 2018). Morphological traits shaped by phenotypic plasticity, provide a broad perspective on the adaptability of species to environmental shifts (Ernande and Dieckmann 2004; Venn 2007; Sommer 2020). However, they become even more informative when analysed alongside other functional traits, which together can reveal deeper ecological insights (Evans et al. 2017; Nolte et al. 2017; Elek, Růžičková, and Ódor 2021).

In ground beetles, wing dimorphism and polymorphism—key indicators of intraspecific phenotypic plasticity—play a critical role in dispersal ability and responses to environmental cues (Ernande and Dieckmann 2004; Belleghem, Roelofs, and Hendrickx 2015; Sommer 2020). By examining wing morphology, this study investigates how habitat changes, particularly forest clearings, influence beetle dispersal strategies and resource use (Venn 2007; Moretti and Legg 2009; Nolte et al. 2017).

In addition, this study evaluates fine‐scale morphological traits to better understand functional responses within beetle assemblage. Using proxy measurements of body traits with species‐specific knowledge, we aim to provide a more comprehensive understanding of ecological roles (Ribera et al. 2001; Barton et al. 2011; Brousseau, Gravel, and Handa 2018). Specifically, this study focuses on three morphological traits: right elytron length, head width and last abdominal sternite (Moretti and Legg 2009; Pedley and Dolman 2014; Langraf et al. 2019).

Right elytron length serves as a proxy for body size, influencing beetle mobility and habitat use (Moretti and Legg 2009; Chiari et al. 2014; Langraf et al. 2018). For traits other than body size, it is important to consider their allometric relationships, as the scaling relative to body size can affect functional interpretations (Kawano 2000; Fox, Cooper, and Hayes 2015; Pélabon et al. 2014). Head width, for instance, is closely related to dietary preference, where larger heads are adapted to consuming hard prey, and smaller heads are suited for soft‐bodied prey (Konuma and Chiba 2007; Laparie et al. 2010). However, this relationship is not always straightforward, as head width may also be influenced by prey availability and the structure of microhabitats (Ribera et al. 1999). Lastly, the last abdominal sternite, particularly in females, serves as an indicator of reproductive potential (Laparie et al. 2010; Evans et al. 2018), although its broader ecological role warrants further investigation (Bolnick et al. 2011; Brousseau, Gravel, and Handa 2018).

To further investigate environmental stress responses, fluctuating asymmetry (FA) is used as a fine‐tuned metric to assess developmental instability under environmental stress (Coda et al. 2016; Papp et al. 2020). FA measures deviations from perfect bilateral symmetry and serves as a reliable indicator of species' responses to habitat disturbances like clear cutting (Palmer 1994; Klingenberg 2019). This study examines FA in relation to environmental factors, including temperature, soil moisture, canopy openness, grass cover, dietary resources and competition, to evaluate developmental disruptions caused by habitat alterations.

The relationship between FA and body size must also be considered, as larger individuals may buffer developmental instability, potentially masking asymmetry (Palmer 2005; Dongen 2006; Møller, Erritzøe, and van Dongen 2018). Controlling for body size ensures that FA accurately reflects developmental instability rather than size variation (Hendrickx, Maelfait, and Lens 2003; Leamy and Klingenberg 2005). Additionally, it is crucial to distinguish FA from other forms of asymmetry, such as measurement error (ME), antisymmetry or effects of wing morphology and sex (Hoffmann and Woods 2001; Vilisics, Sólymos, and Hornung 2005; Elek, Lövei, and Bátki 2014; Budečević et al. 2022).

While previous studies on FA have focused on generalist species in urbanised environments (Elek, Lövei, and Bátki 2014; Papp et al. 2020), this study targets species with narrow ecological tolerances, which may provide deeper insights into stress responses in forest habitats (Moretti and Legg 2009; Pedley and Dolman 2014; Coda et al. 2016). By integrating species composition, morphological traits and FA within a three‐layered framework, this research aims to offer a comprehensive understanding of how forest clearings affect ground beetle assemblages (Ribera et al. 2001; Moretti et al. 2017). This study has three main objectives:

1. Assemblage composition: Using classical multivariate techniques with averaged identity‐based functional traits (ecological and dietary preferences), we examine how the beetle assemblages respond to treatment (control, ecotone and clearing).
2. Morphological traits: We investigate the association between coarse traits (wing morphology) and fine‐scale traits (body size, head width and last abdominal sternite) in relation to treatment conditions.
3. FA: We assess the relationship between environmental factors and FA, considering body size, wing morphology and sex as potential influences.

## 2 Materials and Methods

### 2.1 Study Area and Design

The study was conducted in 2023 at the Podyjí National Park (Southern Moravia, Czech Republic) within the buffer treatment near the village of Lukov (48°52′26.9″ N, 15°53′34.2″ E) at elevations ranging from 385 to 435 m a.s.l. The experimental area, covering 95.1 ha of thermophilous *Quercus petraea* (L.), stands interspersed with *Carpinus betulus* (L.), *Picea abies* (L.) and *Pinus sylvestris* (L.). This area, referred to as ‘Pyramida’, has been used as an experimental area since 1992 to study forest management practices and encompasses a variety of forest types under the conversion process from *Pini‐querceta arenosa* to locally native *Carpini‐querceta* ecosystems (Vrška et al. 2016).

**FIGURE 1:**
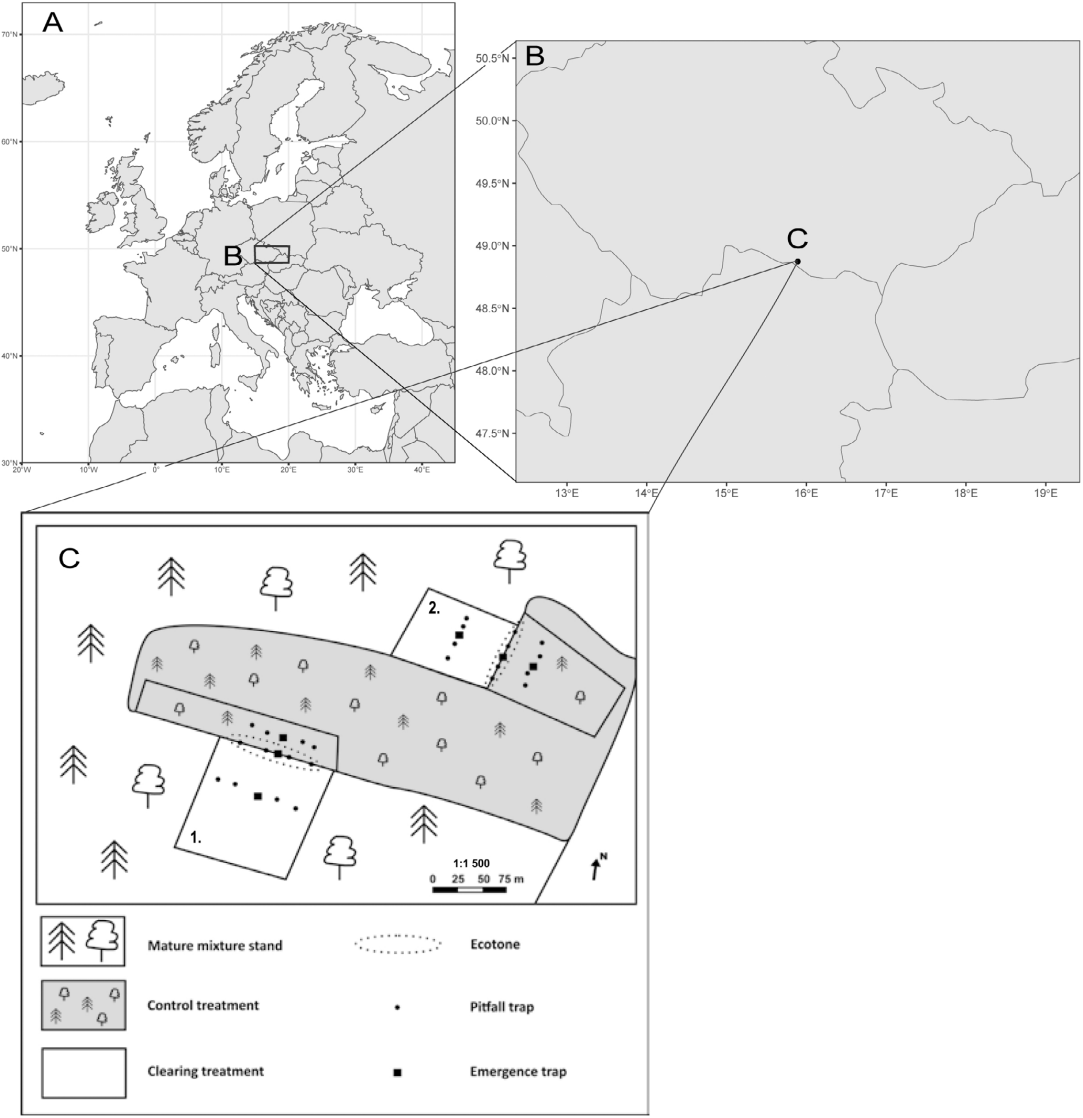
Study area and design—illustration of the geographical setting of the research, highlighting the Czech Republic (A) and pinpointing the study sites within the Pyramida area (C). The boundaries of each treatment (B) are marked by dark line, indicating the periphery of the stands and forestry intervention. The treatments are as follows: clearing—two sparsely populated old‐growth oak clearings; control—younger mixed stands dominated by oak and pine; and ecotone—the transitional zone between the clearing and control treatments.

The study area (Figure 1) consisted of three treatments: clearing, control and ecotone. Each treatment was designed using paired transects:

Clearing treatment involved sparsely populated old‐growth oak areas (*d* = 54 cm; *h* = 27 m; 123 years old), where the last forestry interventions occurred at two sites: The first in 2019 (0.89 ha) and the second in 2021 (0.7 ha), reducing stand density from 0.6 to 0.1 and from 0.4 to 0.1, respectively.

Control treatment consisted of a young mixture stand dominated by oak and pine (*d* = 20 cm; *h* = 12 m; 32 years old), representing an undisturbed comparison to the clearing treatment. The stand density remained at its natural level (1.0), with no intervention.

Ecotone is defined as the transitional zone between the clearing and control treatments. The ecotone captures the gradient of environmental conditions from the open clearing into the denser forest. The transition zone extended between 70 and 85 m in length and varied in width ~20 m, capturing a blend of species and microhabitats from both adjacent areas.

### 2.2 Model Group and Data Collection

At each of the six sites, we established four pitfall traps and one emergence trap, a total of 30 traps across the study. The distance between traps was 10–12 m as per the methodology of Kula (2009) to monitor insect surface activity. To minimise edge effects of outside treatments, the traps were placed at least 15 m from the edges of roads and other stand types as per the guidelines of Helle and Muona (1985) and Niemelä and Spence (1999).

The pitfall traps were installed with their rims slightly elevated above ground level to account for the digging‐in effect, a method referenced by Digweed et al. (1995) and Jiménez‐Carmona, Carpintero, and Reyes‐López (2019). Emergence traps, with surface area of 0.36 m^2^ (Soil Emergence Trap II 60 × 60 cm, black, Ento Sphinx s.r.o., Pardubice, Czech Republic), were centrally positioned among the pitfall traps within each transect to capture emergence of species from the soil, providing a complementary method to the pitfall traps (Marrec et al. 2015) and more robust data (Holland and Reynolds 2003).

These two sampling techniques allowed for a comprehensive investigation of spring activity density and overwintering locations of carabid beetles, which is relevant to the multiscale framework as per the methodology Hanson et al. (2016).

Propylene glycol, diluted in 1:2 ratio, was used as the preservation agent in the traps. The traps remained active throughout the whole study period, with the traps exposed for six periods of 25 days (resulting in 735 trap‐days per year per transect) between mid‐April and mid‐October of 2023. The timing of the traps' exposure was dependent on the vegetation season to ensure coverage of the whole species spectrum, capturing both spring and autumn swarming events (Sapia, Lövei, and Elek 2006).

The area has a heightened level of activity of the ant species *Formica polyctena* (Förster, 1850), which nests in non‐native conifers such as *P. abies* and *P. sylvestris* , occasionally on *Q. petraea* . The species acts as a local source of food competition and may also exert predatory pressure on ground beetles (Iakovlev et al. 2017).

Ecological valence (i.e., relic = specialist and eurytop = generalist) of ground beetles was determined according to Hůrka, Veselý, and Farkač (1996). Data on sex, wing polymorphism type and morphological features were evaluated to assess proxies for body traits and FA. Wing morphology (wing m. for short) was classified according to development into groups (Den Boer et al. 1980), as follows: (i) macropterous (long‐winged morph; M)—these species have fully developed wings and functional flight muscles, allowing them to fly and disperse over large distances; (ii) brachypterous (short‐winged morph; B)—these species possess short wings that may or may not be accompanied by developed flight muscles, generally limiting their ability to fly or disperse; and (iii) apterous individuals (wingless morph; A)—these species are wingless, lacking both wings and flight muscles, which restricts their mobility to ground‐based movement (Schwander and Leimar 2011). After collection in the field, individuals were stored at room temperature in microtubes with 100% propylene glycol. Prior to measurement, individuals were placed on filter paper and dried again at room temperature.

Acronyms for the ground beetle species, along with their full names and corresponding authors, are provided for ease of reference in the ordination diagrams and the evaluation tables detailing identity‐based traits of the species sampled in 2023 (Table S1). Identity‐based traits, including their ecological and dietary preferences, were obtained from the literature and provide insights into the characteristics of the treatments in which these species thrive (Table S1).

### 2.3 Environmental Data

Data on food availability and competition were collected during the sampling of epigeic fauna. The abundance of Collembola was used as a proxy for food availability, while the abundance of *F. polyctena* workers represented competition. Both taxa were captured using pitfall and emergence traps, and their activity levels were measured as the relative abundance of each taxon.

For evaluating the impact of forest management, the following environmental variables were selected: % coverage of herbs in a 25 m^2^ area centred on the pitfall trap (assessed on 13.07.2023); % openness of the canopy above and between traps (eight images per transect) (based on the methodology of Chianucci and Macek 2023); the amount of leaf litter and debris from a 10 × 10 cm area down to the upper layer of ‘A‐horizon’ (kg/m^2^; based on the methodology of Palán, Křeček, and Sato 2018); air temperature measured 15 cm above ground (average for each sampling month) and soil moisture 6 cm below ground (sum of precipitation for the sampling month) using a TMS‐4 datalogger (based on the methodology of Wild et al. 2019).

### 2.4 First Layer: Ground Beetle Assemblage Composition

We used partial canonical correspondence analysis (pCCA) to identify the most critical environmental variables affecting the spatial distribution of ground beetle assemblage composition. Environmental factors with a significant impact on the assemblages were then used in subsequent analysis at third layer to test their correlation with variability in FA. Additionally, the method was utilised to explore the correlation between these significant environmental variables and ground beetles. Month and air temperature served as covariable to control for any systematic or successional changes in species composition that might occur in monitored treatments (i.e., control, ecotone and clearing) irrespective of desired environmental factors. Explanatory variables included treatment, litter and coverage of herbs. Ground beetles were sampled sequentially from mid‐April to mid‐October, therefore the significance of the canonical axes was assessed using a restricted Monte Carlo permutation test. Each permutation was restricted by cyclic shifts maintaining the temporal autocorrelation of individual. Species abundances were log‐transformed, and rare species were downweighted. A forward selection procedure was employed to identify the most parsimonious combination of environmental characteristics explaining the variability in species data. The ordination analysis was conducted using CANOCO 5, as outlined by ter Braak and Šmilauer (2012).

To highlight the importance of the three‐layered statistical analysis, we also calculated community‐weighted means (CWM) of identity‐based functional traits, such as ecological and dietary preferences (Table S2), and correlated them with treatments. Species abundances were log‐transformed, and rare species were downweighted. To calculate variability in identity‐based functional traits, the RaoQ index of functional diversity was applied (Botta‐Dukat 2005). The RaoQ index, like inverse Simpson's species diversity index, expresses the probability that two randomly selected individuals from assemblage will be functionally different (Botta‐Dukat 2005). Both CWM of species and RaoQ index of functional diversity were passively projected into the ordination diagram.

### 2.5 Dataset Preparation for Body Trait Proxies and FA

Before analysing body traits and FA, each separated trait was positioned horizontally in a Petri dish filled with small glass beads of 0.008 mm diameter, and each part was photographed twice under conditions specified in Seifert (2002). The photographs were taken with a Nikon EOS 250D digital camera fitted with a DSLRCC adapter and CMOUNT_1X_SZ mounted on an Olympus SZ 61TR stereomicroscope (0.67 × −4× magnification) at maximum resolution (3840 × 2160 pixels in JPEG format). The images, along with a calibrated scale (ElEla, ElSayed, and Koji 2022), were analysed using the ImageJ software (Schneider, Rasband, and Eliceiri 2012). All subsequent data analyses were conducted in the R environment (v4.3.2; R Development Core Team 2023).

### 2.6 Second Layer: Interaction of Body Trait Proxies With Wing Morphology and Treatment

This layer investigates how coarse traits, such as wing m., interact with fine‐scale body traits. These analyses were conducted on 297 individuals representing 26 ground beetle species. Trait selection and validation included following criteria: low ME; significant variability across treatments; normal variation among individuals; and high ecological relevance. Details about the method used for outlier removal, ME validation and excluded traits are provided in Data S1. From now on, head width was selected based on predefined criteria (Komsta 2022).

#### 2.6.1 Linear Mixed Model for Head Width and Wing Morphology

Before applying the Linear Mixed Model (LMM), a prestep was conducted to examine the effects of wing m. (numeric) on head width while controlling for potential confounding factors, including sex (categorical) and body size. Only significant predictors were retained for the final model, with sex excluded due to its lack of significance.

The LMM was designed to examine the interaction among wing m., body size and treatments, with head width as the primary response variable. Fixed effects included treatment and body size (measured as the length of the right elytron), while wing m. was treated as a numeric covariate. Random effects accounted for the month of collection, which allowed for robust modelling of repeated measurements from multiple sites, and the number of measurement replications per individual, reflecting the nested structure of the study design. The analysis was conducted using the ‘*lme4*’ package (Bates et al. 2015).

#### 2.6.2 Bayesian Generalised Linear Model for Head Width

To establish a robust relationship between head width and studied effects, we extended the model from the LMM step by fitting a Bayesian Generalised Linear Model (BGLM) using the ‘*brm*’ function of the *brms* package (Bürkner 2021). A Gaussian prior distribution was assigned to the model parameters. Computation was performed using four Markov chain Monte Carlo (MCMC) chains with 3000 iterations each, a ‘*max_treedepth*’ of 15 and an ‘*adapt_delta*’ of 0.8.

Model robustness was evaluated using the R‐hat statistic, with all values below 1.0, indicating good convergence. Prediction accuracy of the fitted BGLM was assessed through cross‐validation with the ‘*loo()*’ function of the *loo* package (Vehtari, Gelman, and Gabry 2017). The resulting graphs were created using the *ggplot2* package (Wickham 2016), displaying conditional effects (CI), wing m. on the *x*‐axis and posterior reliability estimates on the *y*‐axis.

### 2.7 Third Layer: Morphological Variability Defined by FA Index

FA was measured on paired structures, including the femurs and tibiae of the forelimbs; the hind femur and metatrochanter; the second, third and fourth antennal segments (hereafter a2, a3 and a4); the length of the second maxillary palpomere (pm2); and the length and width of the third maxillary palpomere (pm3l and pm3w).

Based on data robustness, four species— *Notiophilus rufipes* , *Trechus quadristriatus* , *Poecilus cupreus* and *Bembidion lampros* —met the criteria for reliable FA evaluation, following methodologies outlined by Palmer (1994), Elek, Lövei, and Bátki (2014) and Whalen et al. (2022).

#### 2.7.1 Removal of Unsuitable Traits for the FA Index Dataset

Initial steps to filter out outliers are detailed in Data S1. For subsequent analyses, the following traits were excluded: *T. quadristriatus* (pm2, pm3l and pm3w) and *P. cupreus* (a3).

#### 2.7.2 Body Size Effect Test Using ANOVA

Understanding the dependency of asymmetry on body size is crucial because body size often correlates with developmental stability and environmental adaptability. A larger body size may be a buffer against developmental disruptions that can lead to asymmetry and thus act as an indicator of specimen fitness. By examining the correlation between the absolute differences in trait sides (|*R* − *L*|) and body size (measured as the size of the right elytron), we can determine the necessity to control for body size in further analyses to avoid confounding effects. This step ensures that any observed asymmetry is accurately interpreted as an indicator of developmental instability, rather than simply as a consequence of size variation among individuals. If a significant relationship between trait asymmetry and body size is found, the body size value will then be included as a covariate in the subsequent analyses using LMM and BGLM.

#### 2.7.3 Quantification of Directional Asymmetry and Finalisation of the FA Index Dataset

Our database was recalculated to provide the corrected FA value according to a correction for directional asymmetry (DA) as outlined by Whalen et al. (2022):

$${\mathrm{FA}}_{\text{corrected}}=\sqrt{\left|\left[\frac{R-L}{0.5*\left(L+R\right)}\right]-\left[\frac{\sum \left(\frac{R-L}{0.5*\left(L+R\right)}\right)}{N}\right]\right|}$$

where the first component accounts for individual measurements, and the second component is recalculated as the average of the trait differences, adjusted by the size of the measured individual. The formula takes measured values from both the left and right sides as inputs. The variable ‘*N*’ denotes the total number of individual measurements of a trait. The size difference between the two components was normalised using a quadratic transformation. We used a one‐sample *t*‐test to evaluate the significance of DA, determining whether the mean was significantly different to zero. If the result was significant, the trait was considered biased by DA and excluded from further FA assessment.

#### 2.7.4 Tests of Correlation Between FA Index and Environmental Factors

To understand the relationship between FA and environmental factors, we integrated the evaluation of treatment effects on the FA of individual traits and quantification of the effects of wing morphology, sex and treatment using LMM. For traits with true FA defined by the treatment factor, we used BGLM to explore their associations with environmental factors.

#### 2.7.5 LMM Evaluation of Treatment on FA

We used an LMM to test the relationship between FA and environmental factors. Given the experimental design, where samples were repeatedly collected over time from each plot, the model included collection month and measurement replication within each individual (group) as random effects, with treatment as a fixed effect. The significance of each explanatory factor was analysed using a Wald chi‐squared analysis of variance with the ‘*Anova*’ function from the *car* package (Fox and Weisberg 2019). Outputs from the mixed‐model estimates of averages and differences were described using the ‘*lsmeans*’ function with Tukey correction for p‐value from the *emmeans* package (Russel et al. 2024). This analysis helped us understand how different treatments influenced FA, ensuring that we considered temporal and individual variations.

#### 2.7.6 Simultaneous Quantification of the Effects of Wing Morphology, Sex and Treatment

Next, we assessed the influence of wing m. and sex on the distribution of FA among treatments. This was crucial in the determination of whether the observed asymmetries were due to environmental influences or intrinsic factors like wing m. and sex. The development of the model proceeded through the following steps: (i) the impact of wing m. on FA between sexes was set as the interaction of wing m.: sex, (ii) the effect of wing m. on FA size among treatments was set as the interaction of treatment: wing m. and (iii) the final model included these interactions and tested the relationship of FA distribution between sexes and treatments. We used Levene's test to assess the homogeneity of variances among groups, ensuring an accurate interpretation of the ANOVA results.

#### 2.7.7 Concordance Analysis for Trend Validation Across Multiple FA in Same Species

If true FA was demonstrated in more than two traits, we conducted a test for trend concordance in FA values using Kendall's concordance analysis with Bonferroni correction of p‐values, utilising the ‘*kendall. global*’ function from the *vegan* package (Oksanen et al. 2017). This step was necessary to confirm that the observed FA trends were consistent across multiple traits, providing a robust validation of our findings. The resultant graphs that depict the relationships were created using the *ggplot2* (Wickham 2016) and *ggpubr* packages (Kassambara 2023).

#### 2.7.8 BGLM Between FA Index and Environmental Factors

The trait that met the FA criteria outlined above was thoroughly modelled using a BGLM through the ‘*brm*’ function of the *brms* package (Bürkner 2021). Fixed effects included *F. polyctena* , Collembola, litter, canopy openness, grass coverage, air temperature and soil moisture. Random effects included month, measurement replications for each individual and sex. The a priori distribution for the model parameters was set as a Gaussian distribution. To achieve convergence and good mixing across multiple chains, we configured the model with four chains, each with 4000 iterations, using MCMC. The robustness of the MCMC simulations ensured convergence was evaluated using the *R*‐hat statistic, which remained below a threshold value of 1.2, indicating good model convergence (Gabry and Mahr 2021). Cross‐validation showing a prediction accuracy of the fitted BGLM was done using the ‘*loo()*’ function of the *loo* package (Vehtari, Gelman, and Gabry 2017).

## 3 Results

### 3.1 Comparing Environmental Factors Potentially Important for Shaping Assembly Structure

In 2023, a total of 297 ground beetles, representing 26 species, were recorded across the three treatments. Utilising forward selection (pCCA; *R* ^2^ = 13.7; test on all axes: pseudo‐*F* = 1.5, *p* = 0.018), we identified a significant marginal effect of disturbance and habitat structure characteristics on species assemblages (Table 1). Among the variables tested, the presence of herbs was the most influential factor shaping the assembly structure of thermophilic ground beetles (Table 1), highlighting its critical role in determining species distribution patterns.

**TABLE 1:** A summary calculated by forward selection analysis, with the study month and air temperature set as covariables.

Conditional effects of explanatory variables				
Environmental variables	Explained variation (%)	Pseudo‐*F*	*p*	*p* (adj)*
Control	3.2	2.8	0.004	0.02
Ecotone	1.2	1.0	0.588	0.588
Clearing	2.6	2.2	0.01	0.02
Herbs	2.7	2.4	0.012	0.02
Litter	1.2	1.0	0.256	0.32

*Note:* Explained variation is how much of the variability was explained by the pCCA axes (displayed in Figure 1). The conditional effects of the environmental variables were tested through a Monte‐Carlo with 999 permutations and the *p*‐values (*p*) were corrected by the false discovery rate (*p (adj)).

**FIGURE 2:**
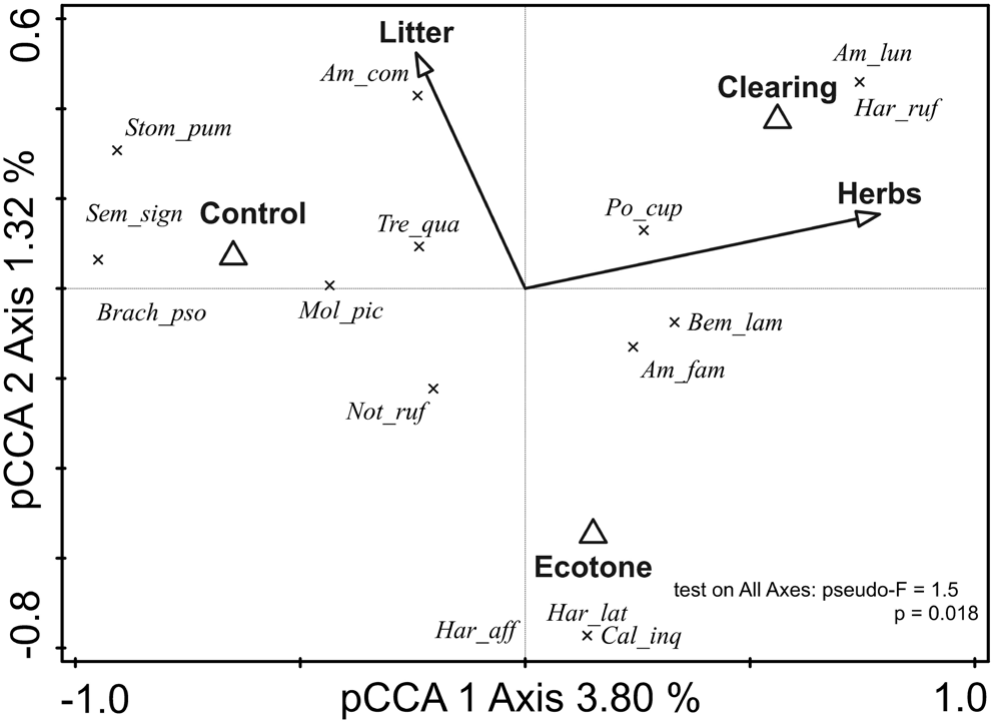
The associations found between the assemblages and environmental variables across the treatments: clearing, control and ecotone, for observations from 2023. The impact of individual environmental variables on these relationships is detailed in Table 1. Within the table, transparent triangles symbolise the treatment, dark arrows with annotations indicate the environmental variables under observation and ‘X’ marks represent species.

An ordination diagram (Figure 2) revealed three distinct species clusters: one associated more with litter presence, another linked to herbaceous cover and the third containing species distributed along the ecotone. The species most frequently found near the centroid of the ordination analysis, representing the most abundant taxa, were *T. quadristriatus* , *B. lampros* , *P. cupreus*, *N. rufipes* and *Molops piceus*. Specifically, *M. piceus* and *T. quadristriatus* exhibited a gradual alignment with increasing vegetation in the upper layer, while *P. cupreus* and *B. lampros* were distributed between the clearing and ecotone.

The relationships between treatments, species distributions and their ecological and dietary preferences, as derived from literature sources, were confronted through passive projections in the ordination analyses (Figures S1–S4). Predatory species predominantly favoured the control treatment, whereas the clearing supported indifferent species that primarily fed on seeds and gastropods. In contrast, the ecotone hosted functionally distinct assemblages, characterised by both ecological and dietary diversity, as evidenced by Rao's quadratic entropy (Figures S2 and S4).

Notably, the dry‐loving *N. rufipes* , which specialises in feeding on Collembola, was frequently captured across all three treatments. However, this species showed no clear dependence on the measured environmental parameters, indicating that its presence may not be strongly influenced by the habitat conditions under study (Figures S1 and S3).

### 3.2 Body Trait Proxy Distribution Between Treatments

**FIGURE 3:**
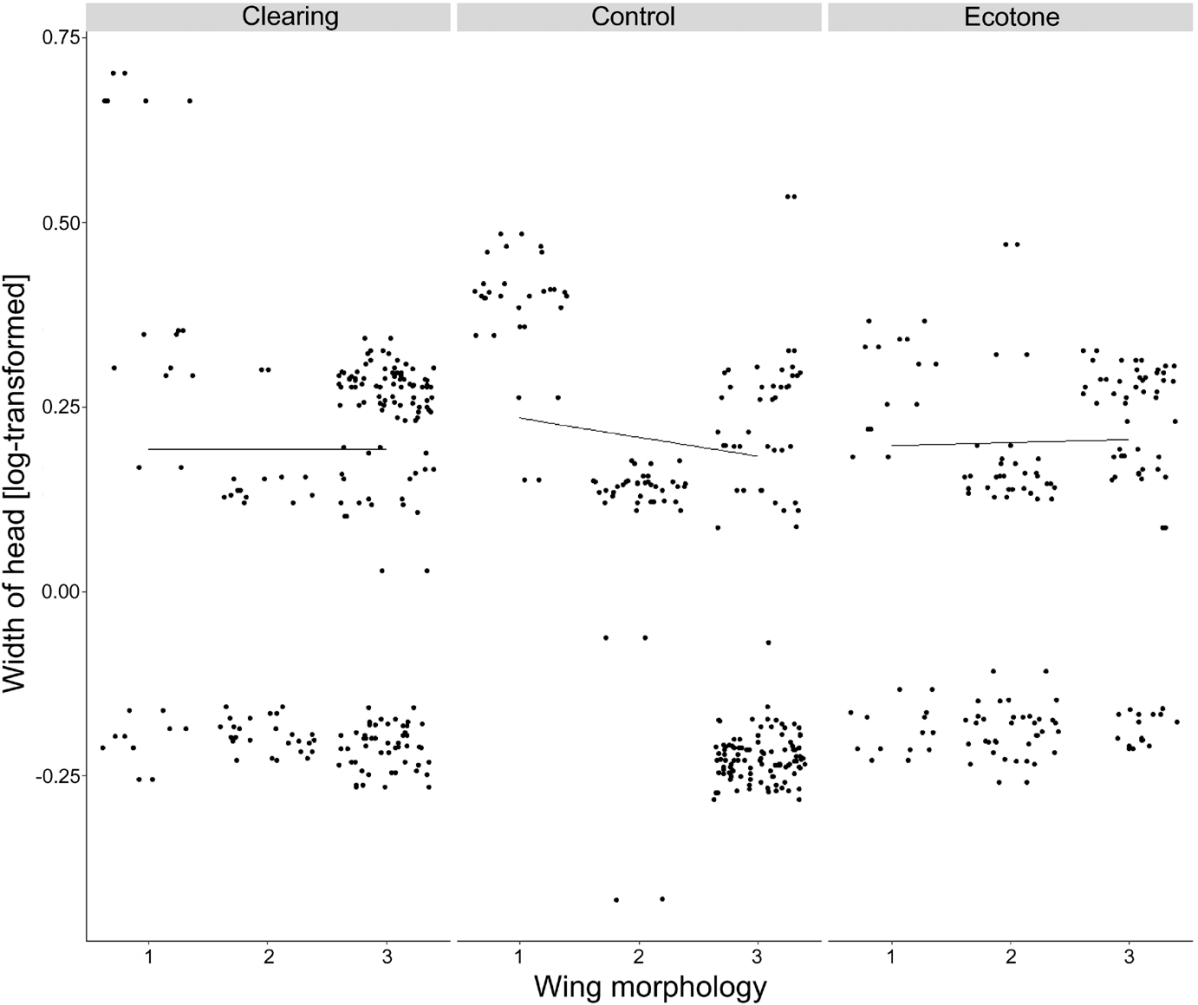
The output from a Bayesian model illustrates the relationship between width of head and wing morphology across different treatments: Clearing, control and ecotone. Black dots represent individual specimen measurements. On the *x*‐axis, abbreviations for wing morphology are as follows: 1—apterous (A), 2—brachypterous (B) and 3—macropterous (M).

The analyses of trait proxy measurements in adult beetle species revealed significant patterns, particularly with respect to head width. Although head width is often linked with dietary preference, this study identified a low‐yet‐significant correlation between wing m. and the control treatment (CI: −0.02 to −0.01) (Table S3 and Figure 3). The absence of small, apterous individuals in the control treatment is supported with reliability of 97.3% (Tables S4, S5 and Figure 3).

### 3.3 Morphological Variability Described by the FA Index and Its Relationship With Environmental Factors

**FIGURE 4:**
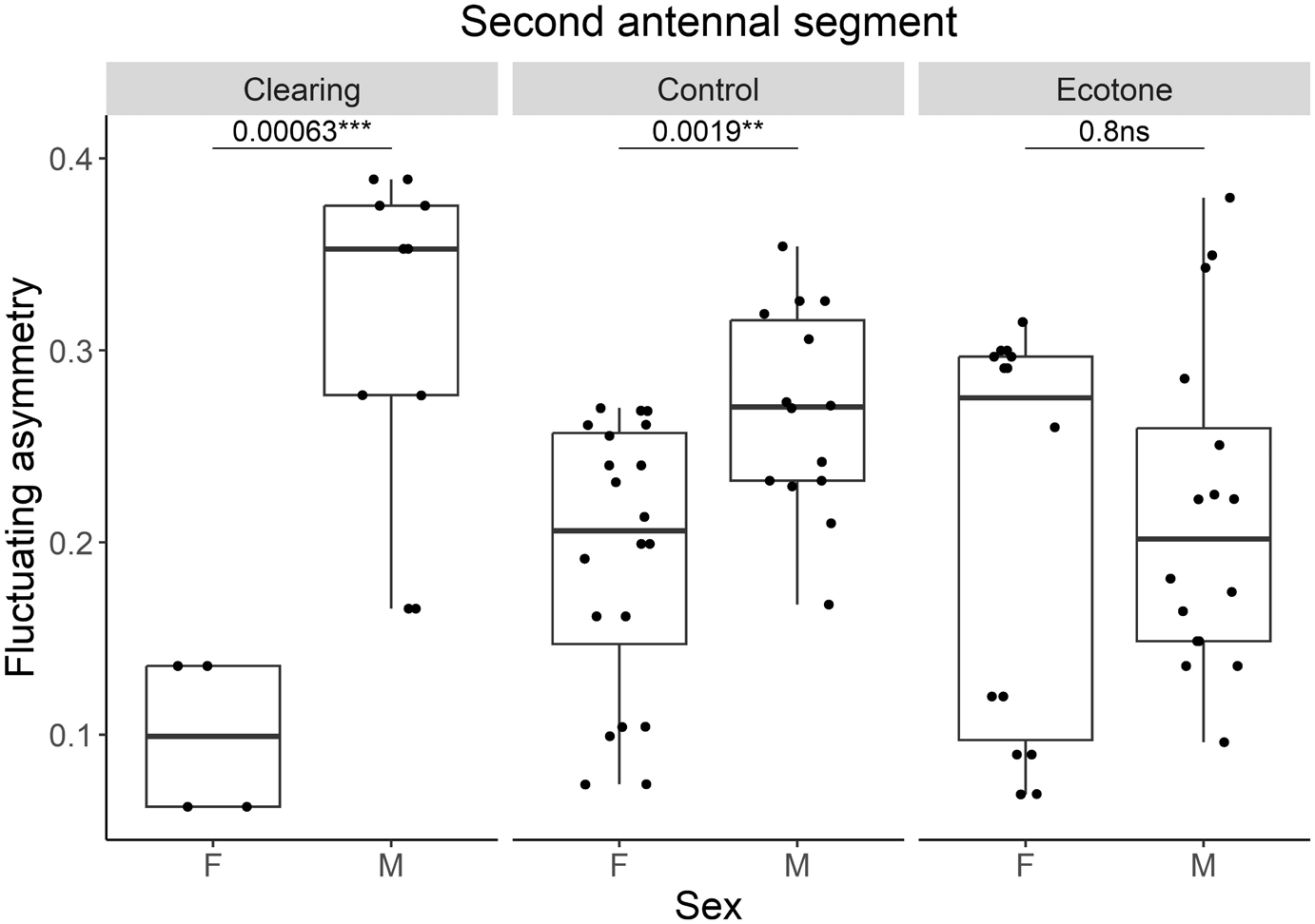
The fluctuating asymmetry of the second antennal segment between sexes (F: female and M: male) in *Notiophilus rufipes*, as shown by the box plot with pairwise comparisons of sexes using ‘tukey_hsd’ test for significance; the dots represent measured values. Adapted from Stočes and Šipoš (2024) with modifications to data presentation.

**FIGURE 5:**
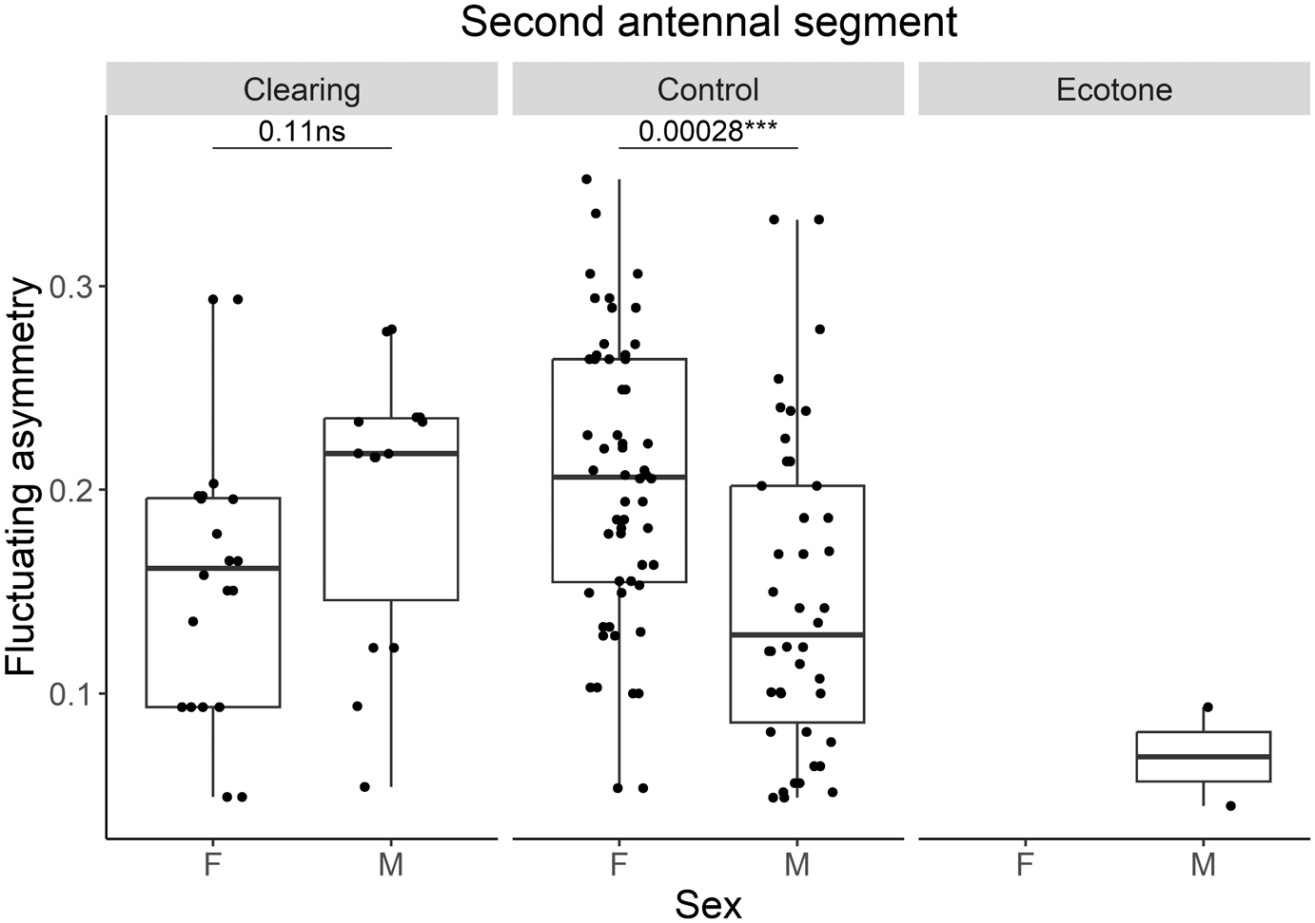
The fluctuating asymmetry of the second antennal segment between sexes (F: female and M: male) in *Trechus quadristriatus* , as shown by the box plot with pairwise comparisons of sexes using ‘tukey_hsd’ test for significance; the dots represent measured values.

From the perspective of FA analyses, species that are locally abundant were studied. These include *N. rufipes* (*n* = 44; *n*_clearing_ = 8, *n*_control_ = 19, *n*_ecotone_ = 17), *T. quadristriatus* (Schrank, 1781) (*n* = 71; *n*_clearing_ = 19, *n*_control_ = 50, *n*_ecotone_ = 2), *P. cupreus* (Linnaeus, 1758) (*n* = 53; *n*_clearing_ = 31, *n*_control_ = 7, *n*_ecotone_ = 12) and *B. lampros* (Herbst, 1784) (*n* = 62; *n*_clearing_ = 29, *n*_control_ = 1, *n*_ecotone_ = 32). *P. cupreus* demonstrated a relationship between the pm3w and DA, which is also influenced by body size (Table S10). Similarly, *B. lampros* (Table S11) exhibits DA in the frontal femur that is not influenced by body size, as evident in macropterous males compared to females (Figure S5). True asymmetry, not affected by any error, was exhibited by a2 in *N. rufipes* (Table S8) and *T. quadristriatus* (Table S9). Other 12 characteristics are not suitable for observing FA, as they are influenced by genetic and additive stress (a3, a4 and frontal femur), antisymmetry (P < L; metatrochanter, pm2, pm3l) and DA (e.g., frontal femur and frontal tibia) (Tables S8 and S9). For *N. rufipes* (analysis of deviance: *p* = 0.028) and *T. quadristriatus* (*p* = 0.021), findings suggest that the effect of treatment on FA is influenced by sex, particularly evident in the control treatment (Tables S12 and S13). Fixed effects of wing m. and body size in LMM do not show significant effects on FA indices, with pseudo‐*R* ^2^ indicating that part of the variability in the models is explained by random effects (Tables S12 and S13). The difference between species lies in their coping mechanism, with specialist *N. rufipes* showing that males are more stressed than females (Figure 4), whereas in the generalist *T. quadristriatus* , the effect is opposite (Figure 5). We depicted the relationship of FA between sexes in treatments by box plots with pairwise comparisons.

**FIGURE 6:**
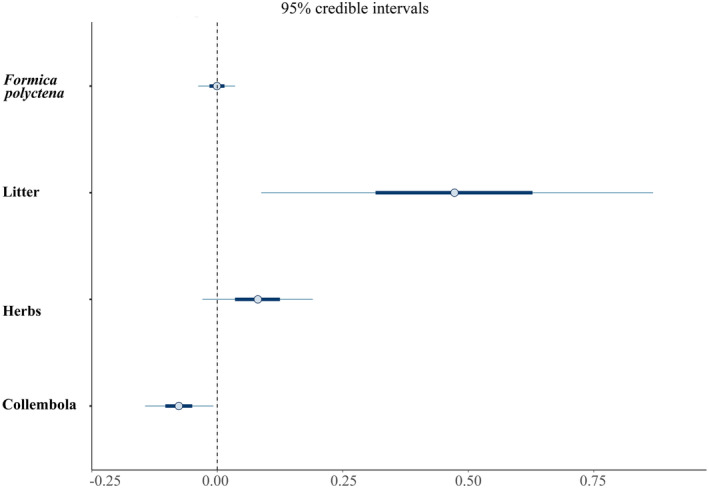
An interval plot of the posterior distribution of coefficients showing the effect size of each explanatory variable on the distribution of FA in the second antennal segment of *Notiophilus rufipes* adults, irrespective of sex.

Pareto *k* diagnostic results of BGLMs confirm the reliability of the estimated posterior distributions of model parameters at 87.2% for the relationship between FA a2 and environmental factors in *N. rufipes* (Table S14) and 87.3% in *T. quadristriatus* (Table S15). In *N. rufipes* , the credible intervals of fixed effects revealed a significant negative association with Collembola (CI: −0.16 to 0.01) and a positive association with litter (CI: 0.01–0.96) on the distribution of FA a2 (Figure 6). Other effects do not show a significant relationship to FA a2 as their confidence intervals include zero (Table S16), thus they are not displayed in Figure 6. In *T. quadristriatus* , no significant associations were found between any environmental factors and FA a2; estimated effects are displayed as in previous case (Table S17).

## 4 Discussion

### 4.1 First Layer: Limitations of Functional Diversity Based on Averaged Ecological and Dietary Traits

In the first layer, ordination diagrams illustrate the influence of human interventions, such as timber harvesting, on species assemblages. In particular, clear cutting alters species composition by changing resource availability and habitat structure (Chen et al. 2021). Mid‐sized predatory, humid‐loving species, as per *M. piceus*, tend to forage in forested habitats, while smaller species, such as *T. quadristriatus* , may dwell in litter and forage as larvae or adults (Harper et al. 2005; Lanta et al. 2020). In contrast, newly created open habitats resulting from clear cutting tend to support hydrophilic and indifferent species that benefit from the formation of grass cover and the altered microclimatic conditions (Magura 2002; Elek, Lövei, and Bátki 2014; Davis et al. 2018; Pinksen et al. 2021).

The ecotone, characterised by species like *Harpalus affinis* , *H. latus* and *Calosoma inquisitor*, enhances functional diversity by fostering transitions between different habitat types (Kuuluvainen 2002; Rischen et al. 2022). This transitional zone also serves as a dispersal pathway for species in mosaic habitats, promoting biodiversity across fragmented landscapes (Yu, Luo, and Zhou 2010; Brigić et al. 2014). Such habitats often support a high degree of species richness, particularly among generalists, which thrive in the ecotonal gradient between clear‐cutting and undisturbed forests (Ribera et al. 1999; Magura et al. 2015).

First‐layer analyses suggest that clearings predominantly support indifferent and hydrophilic beetle assemblages that share common survival strategies (Magura 2002; Harper et al. 2005; Elek, Růžičková, and Ódor 2021). Clear cutting appears to favour opportunistic generalist species such as *B. lampros* , *P. cupreus* and *Amara familiaris* , which quickly respond to environmental cues (Sommer 2020). However, it is important to recognise the limitations of relying solely on averaged traits (Kuuluvainen 2002; Harper et al. 2005; Gossner et al. 2013; Pinksen et al. 2021).

Averaging traits across species can obscure the unique functional responses of individual species, leading to an oversimplification of the community's ecological roles (Barton et al. 2011; Bolnick et al. 2011; Brousseau, Gravel, and Handa 2018). For instance, in the case of the Collembola specialist *N. rufipes* , the ordination analyses provide only a rough indication of environmental influences, likely due to limitations in the sampling method and the masking effects of trait averaging (Elek, Růžičková, and Ódor 2021; Inchausti 2022; Ohlert et al. 2024). Therefore, a more gentle approach that considers species‐specific knowledge is required for understanding the effects of forest management practices (Kuulavainen 2002; Grevé et al. 2018; Tigreros et al. 2023).

### 4.2 Second Layer: Integration of Wing Morphology With Head Width in Habitat Description

In the second layer, we explored how coarse traits, such as wing morphology, interact with fine‐scale body traits, particularly head width, to assess the relationship between ground‐dwelling beetle assemblages and their habitats. This approach is crucial because intraspecific wing plasticity serves as a key indicator of dispersal ability and ecological adaptability in ground beetles, which helps illuminate their responses to environmental changes (Aukema 1995; Barton et al. 2011; Belleghem, Roelofs, and Hendrickx 2015). However, polymorphisms in wing structure can obscure or complicate how traits like head width respond to habitat alterations, particularly in terms of ecological roles and patterns of habitat use (Bolnick et al. 2011; Pélabon et al. 2014; Brousseau, Gravel, and Handa 2018). To address this complexity, it is essential to analyse body traits both independently and in the context of their allometric relationships with body size, wing morphology and sex while considering environmental conditions (Konuma and Chiba 2007; Laparie et al. 2010; Cox et al. 2013; Pélabon et al. 2014).

Our findings suggest that habitat disturbances like clear cutting, which alter microclimatic conditions and resource availability, significantly influence leaf litter and vegetation composition (Harper et al. 2005; Lanta et al. 2020). These changes promote a variety of wing morphs and a wide distribution of head widths in clearings and ecotones. Notably, small apterous beetles were absent from the control treatment, where the more enclosed environment likely favours soil‐nesting species such as *M. piceus* (Brandmayr and Brandmayr 1979). The reduced availability of foraging opportunities may disadvantage smaller apterous beetles, which rely on soft‐bodied, slow‐moving prey (Mitchell 1963; Hanson et al. 2016; Pinksen et al. 2021).

Emergence trap results of small macropterous *T. quadristriatus* specimens suggest that microhabitat changes in the interior may primarily function as sites for larval development rather than for survival and reproduction (Gouix, Zagatti, and Brustel 2009; Hanson et al. 2016; Moretti et al. 2017; Tigreros et al. 2023). This observation is supported by studies indicating that a greater head width provides foraging advantages in ecotonal environments, where diverse prey resources allow beetles to exploit a variety of food sources (Pedley and Dolman 2014; Pinksen et al. 2021). However, not all species may use habitat boundaries primarily for dietary preferences. Some may rely on these boundaries for orientation or dispersal purposes (Riecken and Raths 1996).

These findings underscore the importance of analysing head width as a functional trait alongside wing morphology to gain deeper insights into dietary preferences and ecological roles in response to microhabitat heterogeneity driven by forest management practices (Moretti and Legg 2009; Pedley and Dolman 2014). Moreover, separating the effects of sexes, body size and wing polymorphism is crucial for accurately interpreting the ecological roles species play in different habitat types.

### 4.3 Third Layer: Linking FA Mechanism to Environmental Parameters

In the third layer, after accounting for morphometric measurements of developmental instability (i.e., FA), our analyses revealed distinct habitat conditions associated with phenotypic variability in the four‐most abundant species (Cachada et al. 2013; Moretti et al. 2017; Elek, Lövei, and Bátki 2017). Among these, only *T. quadristriatus* and *N. rufipes* showed significant FA responses, thus forming the main focus of this part of discussion. A modified methodology (enriched with body size and wing morph) was used to examine the relationship between genetic variation and external asymmetry, specifically examining wing polymorphism, body size and previously unexamined patterns of DA between the sexes (Elek, Lövei, and Bátki 2014).

The association of traits with sex and wing morph introduces a potential bias, highlighting the importance of thoroughly evaluating this influence on the distribution of FA (Elek, Lövei, and Bátki 2017; Raisa et al. 2019). Ensuring that traits are not correlated with sex, wing morph or body size is essential, as such correlations can obscure the effects of environmental factors on developmental stability, potentially leading to misinterpretations of functional processes (Vilisics, Sólymos, and Hornung 2005).

Our findings suggest that *P. cupreus* , *B. lampros* , *T. quadristriatus* and *N. rufipes* , while exhibiting phenotypic variability, are still functioning within their tolerance limits (Benítez et al. 2018). The opportunistic macropterous predator *P. cupreus* appears to prioritise foraging over ovipositing in clearings (Marrec et al. 2014). This is indicated both by emergence trapping and the presence of DA in the width of third palpus of the maxillary palpomer, which may offer ecological advantages rather than developmental instability (Dongen 2006; Graham et al. 1998; Bailey et al. 2021; Budečević et al. 2022). *B. lampros* showed trait changes that correlated with sex, partially influenced by wing polymorphism, suggesting a potential link to phenotypic plasticity, possibly reflecting morphological responses to local stressors (Hanson et al. 2016).

FA results further indicate that both *T. quadristriatus* and *N. rufipes* respond to environmental changes through traits like the second antennal segment, which may be less evolutionarily significant (Maryański et al. 2002; Papp et al. 2020). In *N. rufipes* , the second antennal segment likely stems from developmental control mechanisms designed to buffer stress during larval growth (Leamy and Klingenberg 2005; Klingenberg 2019). These mechanisms are crucial for maintaining fitness, particularly for reproductive processes like egg laying in females, where developmental stability is key to reproductive success (Uetz, Papke, and Kilinc 2002; Santangelo et al. 2022).

Conversely, males often exhibit greater morphological variability, reflecting their role in genetic transmission and evolutionarily pressures (Palestrini et al. 2012; Fox et al. 2019; Benítez et al. 2021). The ecotone exhibited notable FA variability among both males and females, supporting the edge effect theory, which facilitates gene flow and reduces isolation (Magura 2002; Nève et al. 2008).

Finally, in the Bayesian model, *N. rufipes* exhibits a link towards stress disturbance, as indicated by its positive association with larval food supply and negative association with leaf litter amount. Adequate food supply helps mitigate environmental stress (Jiménez‐Cortés, Serrano‐Meneses, and Córdoba‐Aguilar 2012), while leaf litter negatively affects the development of the last instar larva, independent of air temperature and soil moisture (Evans et al. 2018). Other environmental factors, like greater activity of the *F. polyctena* ant species (dietary competition), do not have a significant influence on the developmental stability of the larvae. This highlights that species with narrow ecological tolerance can serve as indicators of habitat requirements across both open and forested environments, emphasising their potential as bioindicators of habitat quality (Elek, Růžičková, and Ódor 2021).

In contrast, generalist species, exemplified by *T. quadristriatus* , exhibit high phenotypic plasticity, making it challenging to detect their functional responses to measured environmental factors due to evolutionary mechanisms that buffer developmental processes, ensuring functionality across diverse conditions (Willmore, Young, and Richtsmeier 2007; Lazić et al. 2016). It is also possible that *T. quadristriatus* is responding to environmental factors other than those that have been measured (Gutbrodt et al. 2012). This complexity may explain why previous studies have had difficulty in interpreting results along an urban–rural gradient (e.g., Weller and Ganzhorn 2004; Elek, Lövei, and Bátki 2017; Papp et al. 2020). Despite our predictions, the small sample size limits the robustness of our evidence for developmental pathways and stress in ground beetle assemblages, as noted in Palmer (1994).

### 4.4 Integration of the Three‐Layered Statistical Modelling Approach

This revised multiscale framework empowers researchers and practitioners to: (i) detect subtle functional shifts in microhabitat structures through measured trait variability (Ribera et al. 2001; Brousseau, Gravel, and Handa 2018; Yang, Deng, and Lin 2023); (ii) assess population fitness using intraindividual indicators of developmental instability (Fournier et al. 2015; Papp et al. 2020); and (iii) link species‐specific responses to microhabitat conditions (Lövei and Sunderland 1996; Harper et al. 2005; Moretti and Legg 2009).

By integrating multilevel analysis, this approach enhances research on functional diversity and microhabitat dynamics by capturing critical interactions between species traits and environment (Hanson et al. 2016; Nolte et al. 2017; Elek, Růžičková, and Ódor 2021). It is particularly effective in fragmented or dynamic ecosystems, an example of which is a forest undergoing conversion process (Evans et al. 2018; Chen et al. 2021; Baptista et al. 2024). Although its application requires detailed species‐level data, the framework remains adaptable to diverse ecosystems and Coleoptera taxa. It provides actionable insights for habitat restoration and biodiversity monitoring, serving as a versatile tool for both ecological research and practical conservation applications.

## Author Contributions

**Dominik Stočes:** conceptualization (equal), data curation (lead), formal analysis (equal), funding acquisition (lead), methodology (equal), resources (equal), software (lead), writing – original draft (equal). **Jan Šipoš:** conceptualization (equal), formal analysis (equal), investigation (equal), methodology (equal), validation (equal), writing – original draft (supporting), writing – review and editing (lead).

## Conflicts of Interest

The authors declare no conflicts of interest.

## Supporting information


Data S1.
**Data S2**.

## Data Availability

Datasets used in the present study can be found in Zenodo.org data repository https://doi.org/10.5281/zenodo.11115975.
