# Supplementary material for: Multilevel Analysis of Ground Beetle Responses to Forest Management: Integrating Species Composition, Morphological Traits and Developmental Instability
Source: Ecol Evol. 2025 Jan 21;15(1):e70793. doi: 10.1002/ece3.70793 (PMC11751256; doi:10.1002/ece3.70793)
Supplement: Supplementary file 1 — Data S1. Data S2. [file ECE3-15-e70793-s001.docx]

**Supplementary material**

**Title:** Multilevel analysis of ground beetle responses to forest management: integrating species composition, morphological traits, and developmental instability

**Authors:**

Dominik Stočes^1^, ORCID: 0000-0002-1867-5327

Jan Šipoš^1^, ORCID: 0000-0001-7814-7561

**Affiliations:**

1 – Mendel University in Brno, Faculty of Agronomy, Department of Zoology, Fisheries, Hydrobiology and Apiculture, Zemědělská 1/1665, 613 00 Brno, Czech Republic

**Corresponding authors:** [xstoces@mendelu.cz](mailto:xstoces@mendelu.cz) (D. Stočes)

[I. Supplementary data 1 and 2 2](#_Toc184493438)

[II. Supplementary tables 3](#_Toc184493439)

[II. Supplementary figures 18](#_Toc184493440)

[III. References 23](#_Toc184493441)

# Supplementary data 1 and 2

**Supplementary data 1:** Details about the method used for outlier removal, Measurement Error (ME) validation, and excluded traits in second-layer evaluation.

In our study, body trait proxies were measured to evaluate fine-scale functional predictions obtained from adult beetles and used to gain a deeper understanding of ecological roles. This approach follows the methodology of Evans et al. (2018), which assessed a range of traits including right elytron length (serving as a proxy for body size), head width (linked to dietary preference), wing m. (related to dispersal ability), and the last abdominal sternite (linked to reproductive potential). The interactions were at species level with sex, wing morphs, body size, and treatments.

1. ***Outliers*** were identified based on standardized residuals exceeding an absolute value of 2 and a Cook’s distance threshold calculated as 4 divided by the number of observations. Observations meeting both criteria were excluded to refine the dataset, improve model accuracy, and enhance the fit.
2. ***ANOVA for Measurement Error*** examined the influence of measurement error (ME) on the data for the proxies of body traits to ensure that measurement variability did not skew the results. This was crucial for an assessment of the reliability of our measurements. We used an analysis of variance (ANOVA) to measure ME, incorporating the interaction between individual species variability (ID) and treatment as a fixed factor, and the replication of ME for each individual (Group) as a random effect. This step was essential to differentiate the inherent variability and the environmental effects. Additionally, the proxy of body trait values underwent a log-transformation to allow analysis.
3. ***Linear Mixed Model (LMM)*** analyzing the movement of individuals between treatments based on body size did not provide relevant information (Table S6) and was therefore excluded from the BGLM analysis. However, body size was retained to partition allometric effects in the head width fitting model. Similarly, the LMM for reproduction potential in females between treatments was not significant for the dependent variable and was subsequently removed from further analysis (Table S7).

**Supplementary data 2:** Details about the method used to mitigate the effects of outliers —resulting from Measurement Errors (ME), genetic stress, random events, and directional or antisymmetric deviations—are provided for the third-layer evaluation.

1. ***To provide an accurate assessment of FA***, it was necessary to use the ‘*boxplot.stats*’ function, which is part of the basic version of R. This protocol was applied to both the primary data (R vs. L) and the average values of measured traits ([R - L]/2). If consistency in the outliers was observed across the primary and average data, the individual was excluded, thus eliminating the potential bias arising from these deviations.
2. ***To exclude the influence of directional asymmetry*** (i.e., consistently larger L than R side), we used factorial ANOVA to test if the average size of the trait, measured across all individuals, was significantly different between the L and R sides. Furthermore, traits with high ME were also excluded, as high ME implies that the observed asymmetries might not reflect true developmental instability but rather measurement variability.
3. ***ANOVA for Measurement Error*** was used to quantified ME using an analysis of variance (ANOVA) with body side as a fixed factor (Side) and measurement replications within each individual (Group) as a random factor. If ME (i.e., the ratio of the mean squared error of the fixed factor to the mean squared error of paired measurements) exceeded 15%, the trait was excluded from further statistical analysis (Palmer 1994).
4. ***To eliminate antisymmetry***, we used the *moments* package (Komsta 2022) to assess the degree of skew and the kurtosis of the distribution of measured sides. True and unbiased asymmetry was confirmed using Shapiro's test of normality.

# Supplementary tables

**Table S1:** The acronyms for the ground beetles, as used for the ordination analyses (refer to Fig. 1 in the article). It lists the full names of these species alongside their corresponding authors, adhering to the nomenclature guidelines established by Löbl and Löbl (2018). Bioindication values (R-relic; A-adaptable; E-eurytop) are processed according to Hůrka et al. (1996).

| Species name | Author and year of description | Acronym | Bioindication values |
| --- | --- | --- | --- |
| *Abax parallelepipedus* | Piller et Mitterpacher, 1783 | Ab_par | A |
| *Amara communis* | Panzer, 1797 | Am_com | A |
| *Amara familiaris* | Duftschmid, 1812 | Am_fam | E |
| *Amara lunicollis* | Schödte, 1837 | Am_lun | A |
| *Amara ovata* | Fabricius, 1792 | Am_ova | E |
| *Amara plebeja* | Gyllenhal, 1810 | Am_ple | E |
| *Bembidion lampros* | Herbst, 1784 | Bem_lam | E |
| *Brachinus (B.) psophia* | Audinet-Serville, 1821 | Brach_pso | A |
| *Calathus melanocephalus* | Linnaeus, 1758 | Ca_mel | E |
| *Calosoma inquisitor* | Linnaeus, 1758 | Cal_inq | A |
| *Carabus coriaceus* | Linnaeus, 1759 | Ca_cor | A |
| *Dyschiriodes aeneus* | Dejean, 1825 | Dy_aen | E |
| *Dysohirius globosus* | Herbst, 1784 | Dy_glo | E |
| *Harpalus affinis* | Schrank, 1781 | Har_aff | E |
| *Harpalus atratus* | Latreille, 1804 | Har_atr | A |
| *Harpalus laevipes* | Zetterstedt, 1828 | Har_lae | A |
| *Harpalus latus* | Linnaeus, 1758 | Har_lat | A |
| *Harpalus rubripes* | Duftschmid, 1812 | Har_rub | E |
| *Harpalus rufipalpis* | Sturm, 1818 | Har_ruf | A |
| *Leistus ferrugineus* | Linnaeus, 1758 | Le_fer | E |
| *Loricera (L.) p. pilicornis* | Fabricius, 1775 | Lo_pil | E |
| *Molops piceus* | Panzer, 1793 | Mol_pic | A |
| *Notiophilus rufipes* | Curtis, 1829 | Not_ruf | R |
| *Poecilus cupreus* | Linnaeus, 1758 | Po_cup | E |
| *Pseudophoonus (P.) rufipes* | DeGeer, 1774 | Pse_ruf | E |
| *Pterostichus oblongopunctatus* | Fabricius, 1787 | Pt_obl | A |
| *Semiophonus signaticornis* | Duftschmid, 1812 | Sem_sign | E |
| *Stomis pumicatus* | Panzer, 1796 | Stom_pum | A |
| *Syntomus truncatellus* | Linnaeus, 1761 | Sy_trun | E |
| *Trechus quadristriatus* | Schrank, 1781 | Tre_qua | E |

**Table S2:** Provides supplementary data for species covering the period 2023. This data includes ientity based traits, categorized as follows: ecological preferences (Indifferent, Dry loving, Xerotermic, Humid loving, Hydrophilic), based on the works of Hůrka et al. (1996), Müller-Motzfeld (2004), and Stanovský and Puplán (2006); and dietary preferences (P–predators; G–granivores; eaters focusing on Collembola, Fungi, Opilionides, and Gastropods), referenced from various sources including Lindroth (1949), Thiele (1977), Hůrka (1996), Holland (2002), Turin et al. (2003), Müller-Motzfeld (2004), and Trautner (2017).

| Acronym | Ecological | Dietary |
| --- | --- | --- |
| Ab_par | Hydrophilic | Predator |
| Am_com | Indifferent | Predator; Granivor |
| Am_fam | Humid loving | Granivor |
| Am_lun | Dry loving | Granivor |
| Am_ova | Indifferent | Granivor |
| Am_ple | Indifferent | Collembola |
| Bem_lam | Indifferent | Predator |
| Brach_pso | Humid loving | Granivor |
| Ca_mel | Indifferent | Ganivor, Fungi, Opilionides |
| Cal_inq | Indifferent | Pradotor, Fungi, Opilionides, Gastropods |
| Har_aff | Indifferent | Predator, Granivor |
| Har_atr | Indifferent | Predator, Granivor |
| Har_lae | Indifferent | Predator, Granivor |
| Har_lat | Indifferent | Predator, Granivor |
| Har_rub | Dry loving | Granivor |
| Har_ruf | Indifferent | Predator, Granivor |
| Le_fer | Indifferent | Collembola |
| Lo_pil | Indifferent | Predator, Collembola |
| Mol_pic | Humid loving | Predator |
| Not_ruf | Dry loving | Collembola |
| Po_cup | Indifferent | Predator, Gatstropods |
| Pse_ruf | Indifferent | Predator, Gatstropods |
| Sem_sign | Dry loving | Predator |
| Stom_pum | Humid loving | Predator |
| Sy_trun | Dry loving | Predator |
| Tre_qua | Indifferent | Predator; Collembola |

**Table S3:** Credible intervals of dependent variable width of head with wing morphology (wing m. for short; set as nummeric) set in with population- and group-level effects of the Bayesian Model; the significant result is highlighted in bold

| Population-level effects | | | | | |
| --- | --- | --- | --- | --- | --- |
|  | Estimate | Estimated error | l-95% CI | u-95% CI | Rhat |
| Intercept | 0.29 | 0.03 | 0.23 | 0.36 | 1.00 |
| Right elytron | 0.17 | 0.03 | 0.10 | 0.23 | 1.00 |
| Control | 0.03 | 0.01 | 0.01 | 0.04 | 1.00 |
| Ecotone | 0.00 | 0.01 | -0.01 | 0.01 | 1.00 |
| Wing m. | -0.00 | 0.00 | -0.01 | 0.01 | 1.00 |
| **Control: Wing m.** | **-0.02** | **0.00** | **-0.02** | **-0.01** | **1.00** |
| Ecotone: Wing m. | 0.00 | 0.00 | -0.01 | 0.01 | 1.00 |
| Group-level effects | | | | | |
| Month | 0.00 | 0.00 | 0.00 | 0.01 | 1.00 |
| Specimen | 0.11 | 0.02 | 0.08 | 0.14 | 1.00 |
| Specimen: Group | 0.02 | 0.00 | 0.02 | 0.02 | 1.00 |

**Table S4:** Extracted posterior estimates of the population-level ('fixed') effects

|  | Estimate | Est.Error | Q2.5 | Q97.5 |
| --- | --- | --- | --- | --- |
| Intercept | 0.290320065 | 0.034087117 | 0.225343905 | 0.360896846 |
| Right elytron | 0.167489628 | 0.032414410 | 0.103753342 | 0.231778122 |
| Control | 0.026260672 | 0.007919703 | 0.011119055 | 0.042001256 |
| Ecotone | 0.001624271 | 0.006578951 | -0.011036663 | 0.014544744 |
| Wing m. | -0.001345447 | 0.003850013 | -0.008893137 | 0.006349749 |
| Control: Wing m. | -0.015614388 | 0.004548029 | -0.024673468 | -0.006850386 |
| Ecotone: Wing m. | 0.002215300 | 0.004126786 | -0.005893687 | 0.010427038 |

**Table S5:** Percentage evaluation of the reliability of the estimated posterior distributions of the parameters

| Pareto *k* diagnostic values: | | | | |
| --- | --- | --- | --- | --- |
|  |  | Count | % | Min_ESS |
| (-Inf; 0,5] | Good | 361 | 60.8 | 970 |
| (0,5; 0,7] | OK | 217 | 36.5 | 244 |
| (0,7; 1] | Bad | 10 | 1.7 | 79 |
| (1; Inf) | Very bad | 6 | 1.0 | 2 |

**Table S6:** Results of the fixed effects of the Linear Mixed Model on the dependent variable body size (AIC = -4521.19; BIC -4477.32) with marginal R^2^ = 0.00 and conditional R^2^ = 1.00

| Fixed effects: | | | | | |
| --- | --- | --- | --- | --- | --- |
|  | Estimates | Standardised error | t values | Degrees of freedom | P–values^1^ |
| (Intercept) | 0.69 | 0.05 | 12.92 | 31.27 | 0.00 |
| Control | 0.02 | 0.02 | 0.89 | 263.23 | 0.37 |
| Ecotone | 0.00 | 0.01 | 0.22 | 218.34 | 0.83 |
| Wing m. | 0.00 | 0.01 | 0.40 | 264.22 | 0.69 |
| Control: Wing m. | -0.01 | 0.01 | -0.95 | 262.74 | 0.34 |
| Ecotone: Wing m. | 0.00 | 0.01 | -0.58 | 261.34 | 0.56 |
| Random effects: | | | | | |
| Standardised deviations | | | | | |
| Month | 0.01 |  |  |  |  |
| Specimen | 0.28 |  |  |  |  |
| Specimen: Group | 0.04 |  |  |  |  |
| Residual | 0.00 |  |  |  |  |
| Grouping variables: | | | | | |
|  | groups | ICC |  |  |  |
| Specimen: Group | 296 | 0.02 |  |  |  |
| Specimen | 29 | 0.98 |  |  |  |
| Month | 5 | 0.00 |  |  |  |

^1^P–values calculated using Satterthwaite d.f.

**Table S7:** Fitted results of the fixed effects of the Linear Mixed Model on the dependent vairable last abdominal sternite (AIC = -3868.99; BIC -3820.73) with marginal R^2^ = 0.24 and conditional R^2^ = 1.00

| Fixed effects: | | | | | |
| --- | --- | --- | --- | --- | --- |
|  | Estimates | Standardised error | t values | Degrees of freedom | P–values^1^ |
| (Intercept) | -0.32 | 0.06 | -5.36 | 89.78 | 0.00 |
| Right elytron | 0.48 | 0.07 | 7.34 | 329.49 | 0.00 |
| Control | 0.01 | 0.01 | 0.48 | 263.01 | 0.63 |
| Ecotone | 0.01 | 0.01 | 0.94 | 242.40 | 0.35 |
| Wing m. | -0.01 | 0.01 | -0.98 | 260.61 | 0.33 |
| Control: Wing m. | 0.01 | 0.01 | -0.97 | 265.43 | 0.33 |
| Ecotone: Wing m. | -0.01 | 0.02 | -0.43 | 261.78 | 0.67 |
| Random effects: | | | | | |
| Standardised deviations | | | | | |
| Month | 0.00 |  |  |  |  |
| Specimen | 0.20 |  |  |  |  |
| Specimen: Group | 0.06 |  |  |  |  |
| Residual | 0.00 |  |  |  |  |
| Grouping variables: | | | | | |
|  | groups | ICC |  |  |  |
| Specimen: Group | 296 | 0.07 |  |  |  |
| Specimen | 29 | 0.93 |  |  |  |
| Month | 5 | 0.00 |  |  |  |

^1^P–values calculated using Satterthwaite d.f.

**Table S8:** Summary of results for 10 measured morphological traits of *Notiophilus rufipes*. The sequence of recorded results corresponds with the procedure outlined in subsection 2.7, 'Statistical Evaluation of the Third Layer: Morphological Variability'; significant values are in bold

**Abbreviations: a2** second antennal segment; **a3** third antennal segment; **a4** fourth antennal segment; **pm2** length of second palpomer of the maxillary palpus; **pm3l** length of third palpomer of the maxillary palpus; **pm3w** width of third palpomer of the maxillary palpus

**Table S9:** Summary of results for 13 measured morphological traits of *Trechus quadristriatus*. The sequence of recorded results corresponds with the procedure outlined in subsection 2.7, 'Statistical Evaluation of the Third Layer: Morphological Variability'; significant values are in bold

**Abbreviations: a2** second antennal segment; **a3** third antennal segment; **a4** fourth antennal segment; **pm2** length of second palpomer of the maxillary palpus; **pm3l** length of third palpomer of the maxillary palpus; **pm3w** width of third palpomer of the maxillary palpus; **pl1** length of first palpomer of the labialis palpus; **pl2l** length of second palpomer of the labialis palpus; **pl2w** width of second palpomer of the palpomer of the labialis palpus

**Table S10:** Summary of results for 13 measured morphological traits of *Poecilus cupreus*. The sequence of recorded results corresponds with the procedure outlined in subsection 2.7, 'Statistical Evaluation of the Third Layer: Morphological Variability'; significant values are in bold

**Abbreviations: a2** second antennal segment; **a3** third antennal segment; **a4** fourth antennal segment; **pm2** length of second palpomer of the maxillary palpus; **pm3l** length of third palpomer of the maxillary palpus; **pm3w** width of third palpomer of the maxillary palpus; **pl1** length of first palpomer of the labialis palpus; **pl2l** length of second palpomer of the labialis palpus; **pl2w** width of second palpomer of the palpomer of the labialis palpus

**Table S11:** Summary of results for 10 measured morphological traits of *Bembidion lampros*. The sequence of recorded results corresponds with the procedure outlined in subsection 2.7, 'Statistical Evaluation of the Third Layer: Morphological Variability'; significant values are in bold

**Abbreviations: a2** second antennal segment; **a3** third antennal segment; **a4** fourth antennal segment; **pm2** length of second palpomer of the maxillary palpus; **pm3l** length of third palpomer of the maxillary palpus; **pm3w** width of third palpomer of the maxillary palpus

**Table S12:** Results of the fixed effects of the Linear Mixed Model on the dependent vairable second antennal segment of *Notiophilus rufipes* (AIC = -178.17; BIC -152.25) with marginal R^2^ = 0.26 and conditional R^2^ = 0.93; the main significant value is highlighted in in bold

| Fixed effects: | | | | | |
| --- | --- | --- | --- | --- | --- |
|  | Estimates | Standardised error | t values | Degrees of freedom | P–values^1^ |
| (Intercept) | -0.01 | 0.60 | -0.01 | 31.00 | 0.99 |
| Wing m. | -0.03 | 0.03 | -1.07 | 31.00 | 0.29 |
| Right elytron | 0.22 | 0.98 | 0.23 | 31.00 | 0.82 |
| Control | 0.11 | 0.07 | 1.62 | 31.00 | 0.12 |
| Ecotone | 0.12 | 0.07 | 1.67 | 31.00 | 0.11 |
| Sex (male) | 0.23 | 0.07 | 3.11 | 31.00 | 0.00 |
| **Control: Sex** | **-0.16** | **0.09** | **-1.91** | **31.00** | **0.06** |
| Ecotone: Sex | -0.23 | 0.09 | -2.68 | 31.00 | 0.01 |
| Random effects: | | | | | |
| Standardised deviations | | | | | |
| Group | 0.08 |  |  |  |  |
| Month | 0.00 |  |  |  |  |
| Residual | 0.03 |  |  |  |  |
| Grouping variables: | | | | | |
|  | groups | ICC |  |  |  |
| Group | 39 | 0.90 |  |  |  |
| Month | 5 | 0.00 |  |  |  |

^1^P–values calculated using Satterthwaite d.f.

**Table S13:** Results of the fixed effects of the Linear Mixed Model on the dependent vairable second antennal segment of *Trechus quadristriatus* (AIC = -527.20; BIC -501.12) with marginal R^2^ = 0.26 and conditional R^2^ = 0.93; the significant value is highlighted in bold

| Fixed effects: | | | | | |
| --- | --- | --- | --- | --- | --- |
|  | Estimates | Standardised error | t values | Degrees of freedom | P–values^1^ |
| (Intercept) | 0.35 | 0.23 | 1.50 | 31.00 | 0.14 |
| Right elytron | -0.33 | 0.44 | -0.75 | 31.00 | 0.46 |
| Control | 0.04 | 0.03 | 1.58 | 31.00 | 0.12 |
| Ecotone | -0.16 | 0.08 | -2.05 | 31.00 | 0.05 |
| Sex (male) | 0.04 | 0.04 | 1.08 | 31.00 | 0.29 |
| **Control: Sex** | **-0.10** | **0.04** | **-2.30** | **31.00** | **0.02** |
| Random effects: | | | | | |
| Standardised deviations | | | | | |
| Group | 0.08 |  |  |  |  |
| Month | 0.00 |  |  |  |  |
| Residual | 0.03 |  |  |  |  |
| Grouping variables: | | | | | |
|  | groups | ICC |  |  |  |
| Group | 39 | 0.90 |  |  |  |
| Month | 5 | 0.00 |  |  |  |

^1^P–values calculated using Satterthwaite d.f.

**Table S14:** Percentage evaluation of the reliability of the estimated posterior distributions of the parameters of the Bayesian Generalized Linear Model used for *Notiophilus rufipes*

| Pareto *k* diagnostic values: | | | | |
| --- | --- | --- | --- | --- |
|  |  | Count | % | Min_ESS |
| (-Inf; 0,5] | (Good) | 30 | 38.5 | 1249 |
| (0,5; 0,7] | OK | 38 | 48.7 | 263 |
| (0,7; 1] | (Bad) | 8 | 10.3 | 36 |
| (1; Inf) | (Very bad) | 2 | 2.6 | 6 |

**Table S15:** Percentage evaluation of the reliability of the estimated posterior distributions of the parameters of the Bayesian Generalized Linear Model used for *Trechus quadristriatus*

| Pareto *k* diagnostic values: | | | | |
| --- | --- | --- | --- | --- |
|  |  | Count | % | Min_ESS |
| (-Inf; 0,5] | (Good) | 72 | 53.5 | 1382 |
| (0,5; 0,7] | OK | 45 | 33.6 | 208 |
| (0,7; 1] | (Bad) | 13 | 9.7 | 17 |
| (1; Inf) | (Very bad) | 4 | 3.0 | 3 |

**Table S16:** Credible intervals of dependent variable fluctuating asymmetry of second antennal segment of *Notiophilus rufipes* with population- and group-level effects; the significant results are highlighted in bold

| Population-level effects: | | | | | |
| --- | --- | --- | --- | --- | --- |
|  | Estimate | Estimated error | l-95% CI | u-95% CI | Rhat |
| Intercept | 0.33 | 0.73 | -0.98 | 1.94 | 1.00 |
| *Formica polyctena* | 0.00 | 0.02 | -0.05 | 0.04 | 1.00 |
| **Litter** | **0.47** | **0.24** | **0.01** | **0.96** | **1.00** |
| **Collembola** | **-0.08** | **0.04** | **-0.16** | **0.01** | **1.00** |
| Openness | -0.18 | 0.18 | -0.54 | 0.16 | 1.00 |
| Herbs | 0.08 | 0.07 | -0.05 | 0.21 | 1.00 |
| Temperature | -0.02 | 0.03 | -0.08 | 0.02 | 1.00 |
| Soil moisture | 0.26 | 0.18 | -0.10 | 0.61 | 1.00 |
|  |  |  |  |  |  |
| Group-level effects: | | | | | |
| sd(Intercept) | 0.09 | 0.01 | 0.07 | 0.12 | 1.00 |
| Month | 0.07 | 0.09 | 0.00 | 0.31 | 1.00 |
| Sex | 0.26 | 0.29 | 0.01 | 1.08 | 1.01 |
| Group | 0.09 | 0.01 | 0.07 | 0.12 | 1.00 |

**Table S17:** Credible intervals of dependent variable fluctuating asymmetry of second antennal segment of *Trechus quadristriatus* with population- and group-level effects

| Population-level effects: | | | | | |
| --- | --- | --- | --- | --- | --- |
|  | Estimate | Estimated error | l-95% CI | u-95% CI | Rhat |
| Intercept | -0.76 | 1.56 | -3.67 | 2.70 | 1.00 |
| *Formica polyctena* | 0.01 | 0.03 | -0.05 | 0.06 | 1.00 |
| Litter | 0.06 | 0.08 | 0.10 | 0.23 | 1.00 |
| Collembola | -0.02 | 0.03 | -0.08 | 0.04 | 1.00 |
| Openness | -0.01 | 0.06 | -0.13 | 0.11 | 1.00 |
| Herbs | 0.01 | 0.03 | -0.05 | 0.06 | 1.00 |
| Temperature | 0.67 | 1.19 | -2.00 | 2.86 | 1.00 |
| Soil moisture | 0.05 | 0.16 | -0.25 | 0.41 | 1.00 |
|  |  |  |  |  |  |
| Group-level effects: | | | | | |
| sd(Intercept) | 0.08 | 0.01 | 0.06 | 0.09 | 1.00 |
| Month | 0.08 | 0.09 | 0.00 | 0.33 | 1.00 |
| Sex | 0.22 | 0.24 | 0.01 | 0.91 | 1.01 |
| Group | 0.08 | 0.01 | 0.06 | 0.09 | 1.00 |

# Supplementary figures

**
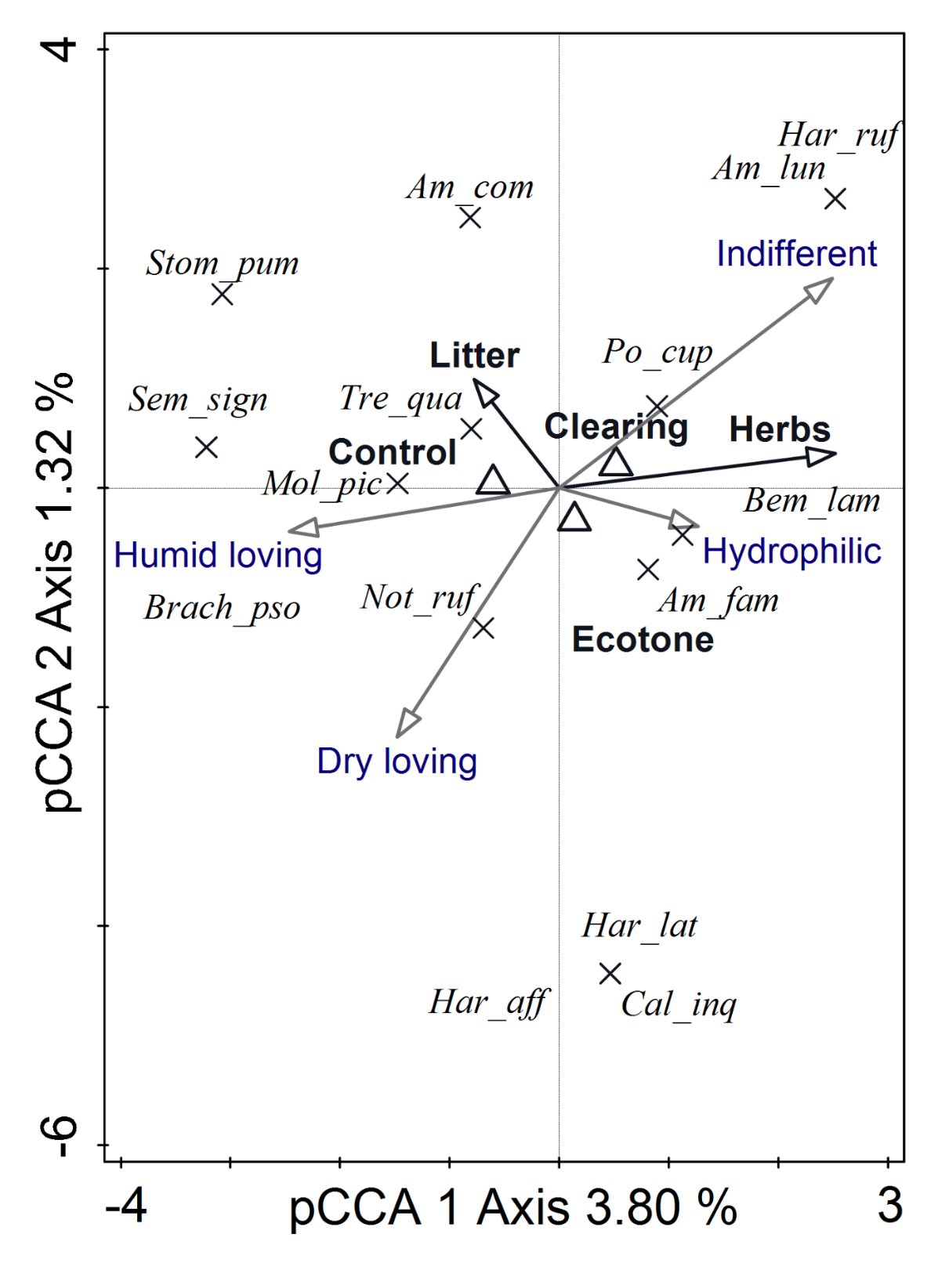
**

**Fig. S1:** pCCA elucidates the connections between identity-based ecological preferences and their distribution across treatments. For visual representation, the diagram employs transparent triangles to denote the treatments, 'X' marks for species and a grey arrows with an annotations to indicate ecological preferences.

**
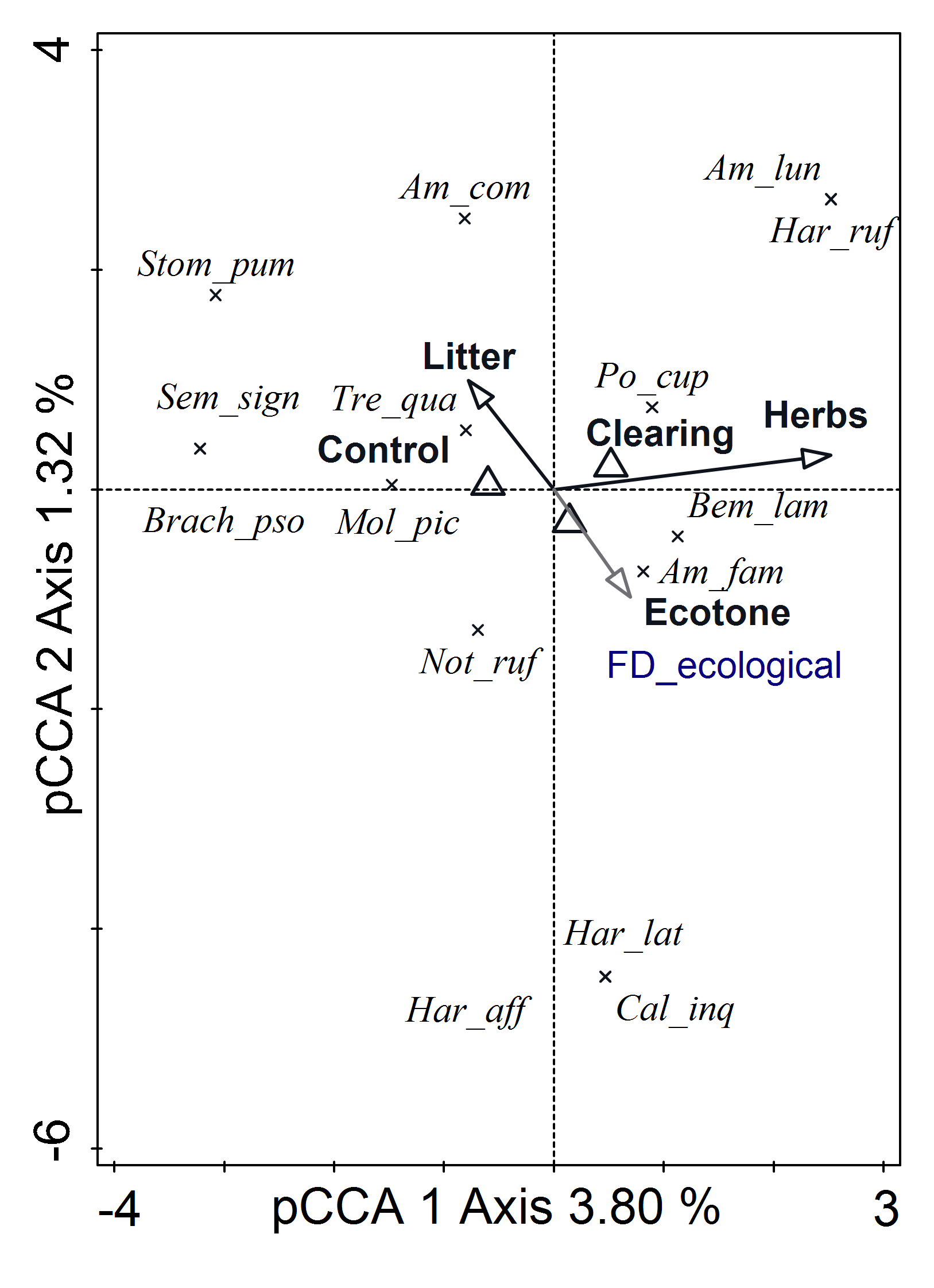
Fig. S2:** The pCCA, focusing on ecological preferences, highlights the functional diversity and its role in explaining the niche bioindicative ecological utilization rates across treatments, all monitored during the 2013 period. The diagrammatic representation uses transparent triangles to denote the treatments, 'X' marks for species, and a grey arrow with an annotation to indicate the functional diversity as determined by the RaoQ index.

**
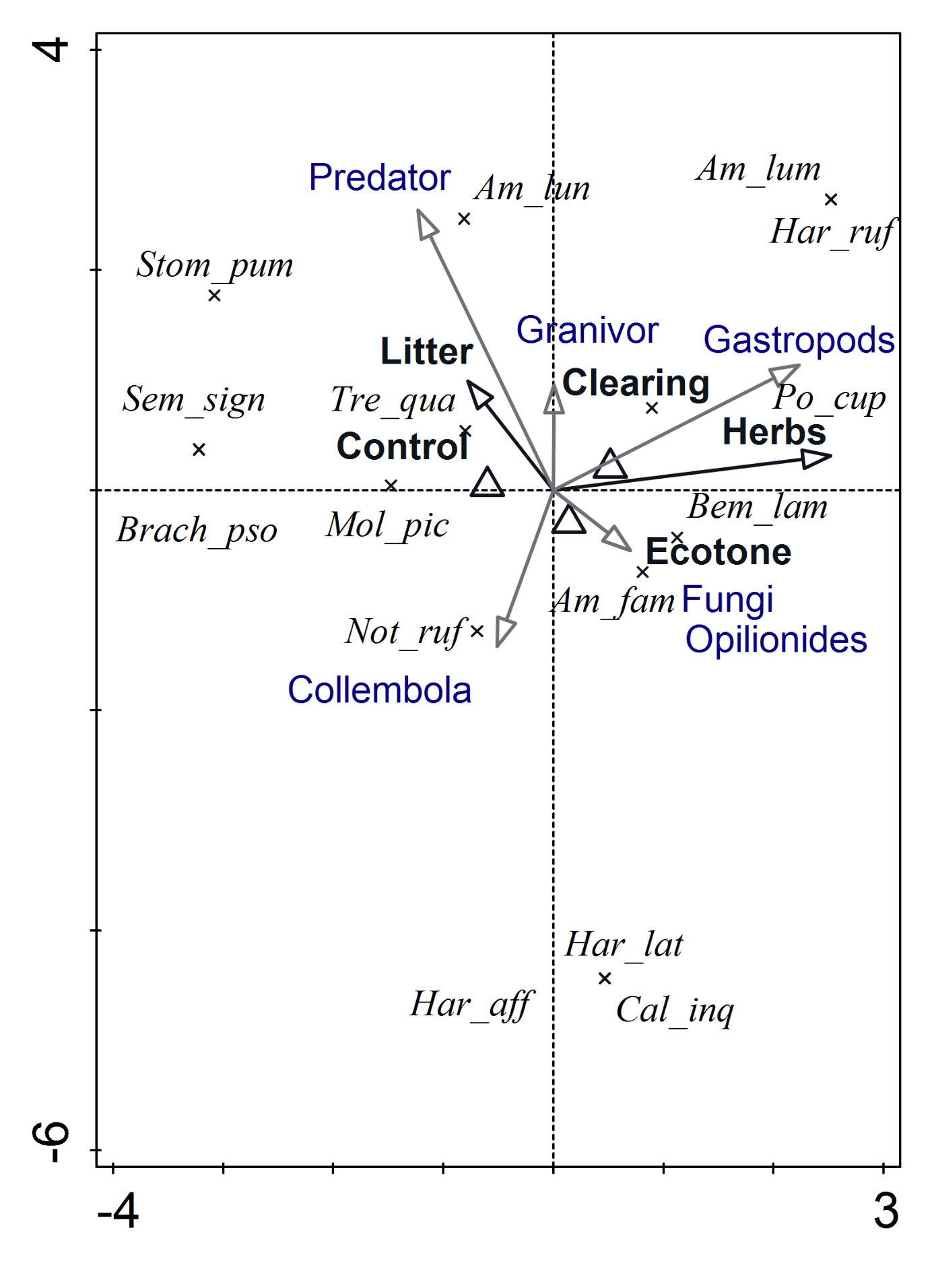
Fig. S3:** pCCA elucidates the connections between identity-based dietary preferences and their distribution across treatments. For visual representation, the diagram employs transparent triangles to denote the treatments, 'X' marks for species and a grey arrows with an annotations to indicate ecological preferences.

**
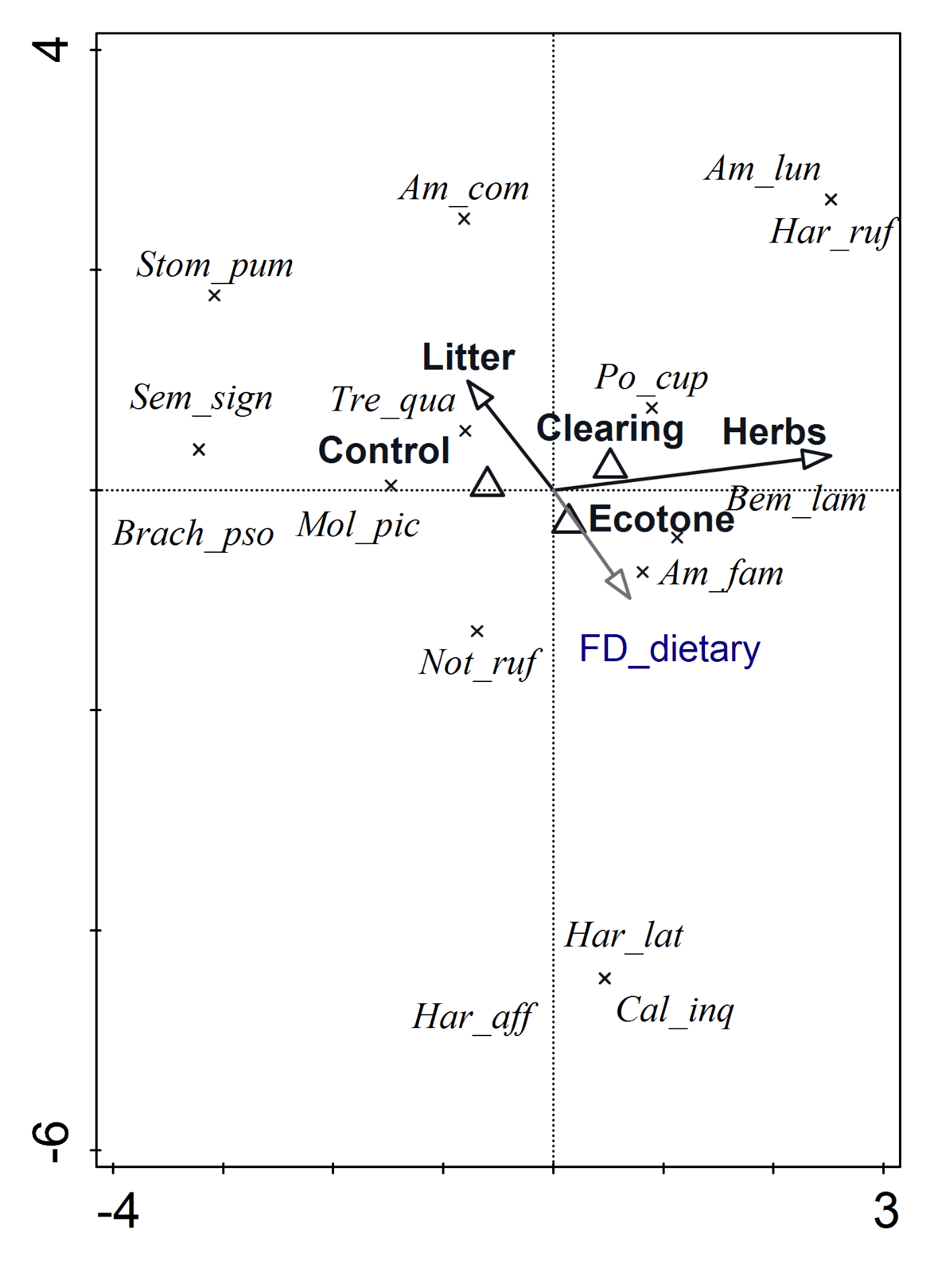
Fig. S4:** The pCCA, focusing on dietary preferences, highlights the functional diversity and its role in explaining the niche bioindicative dietary utilization rates across treatments, all monitored during the 2013 period. The diagrammatic representation uses transparent triangles to denote the treatments, 'X' marks for species, and a grey arrow with an annotation to indicate the functional diversity as determined by the RaoQ index.

**
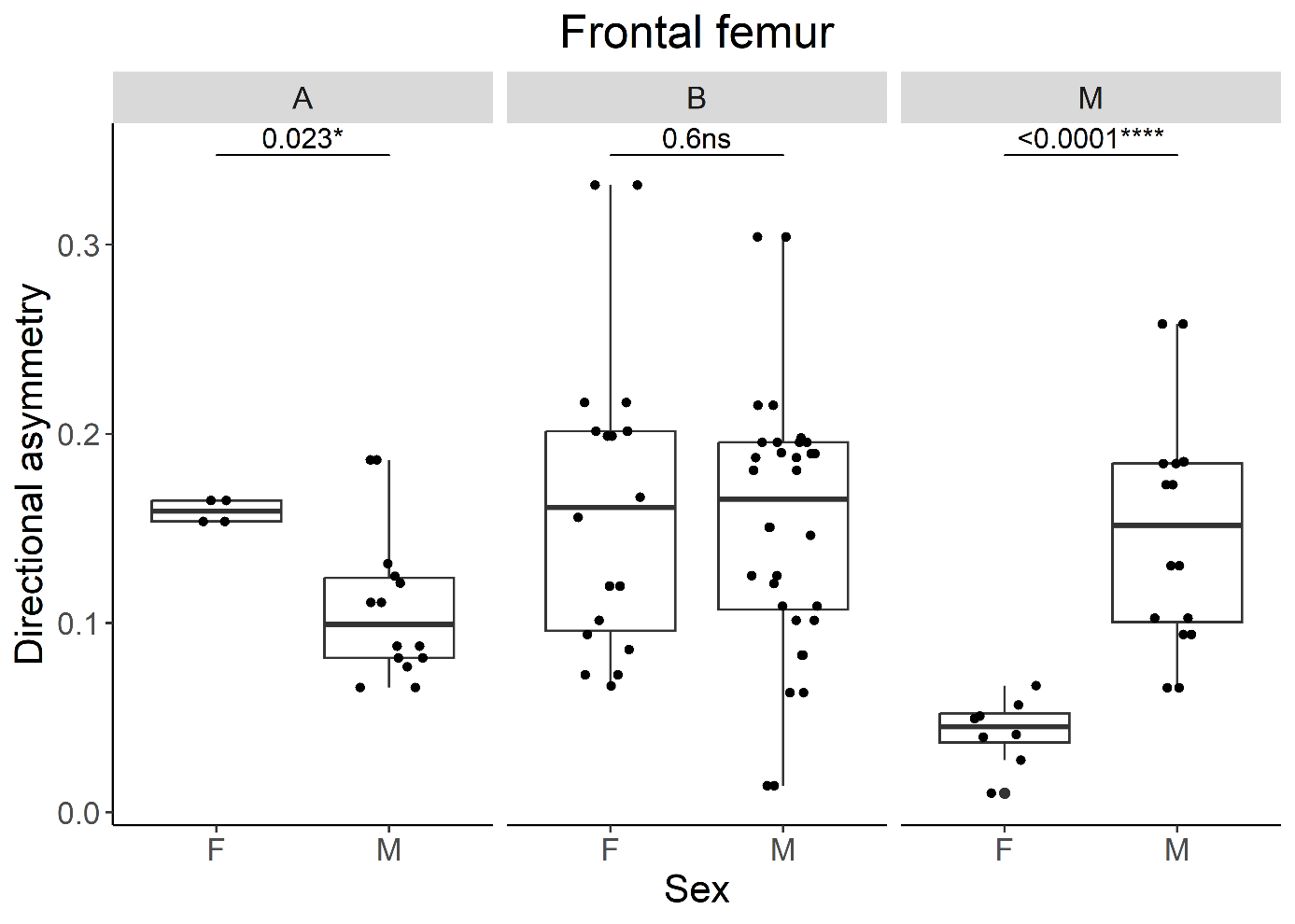
Fig. S5:** Directional asymmetry of frontal femur between sexes (F: female; M: male) in observed wing morphology types of *Bembidion lampros*, as shown by box plot with pair wise comparisons of sexes using ‘tukey_hsd’ test for significance; the dots represent measured values

# References

Evans MJ, Banks SC, Barton PS, Davies KF, Driscoll DA (2018) A Long-Term Habitat Fragmentation Experiment Leads to Morphological Change in a Species of Carabid Beetle. Ecological Entomology. 43, pp 282–293. https://doi.org/10.1111/een.12498.

Holland JM (2002) The agroecology of carabid beetles. Andover: Intercept Limited, pp 356. ISBN: 1898298769.

Hůrka K (1996) Carabidae České a Slovenské republiky. [Carabidae of the Czech and Slovak republics.] Zlín, Kabourek, pp 565. ISBN 80-901466-2-7.

Hůrka K, Veselý P, Farkač J (1996) Využití střevlíkovitých (Coleoptera: Carabidae) k indikaci kvality prostředí; Die Nutzung der Laufkäfer (Coleoptera: Carabidae) zur Indikation der Umweltqualität. Klapalekiana. 32, pp 15–26. 11-15-Hejkal.pdf (entolisty.cz).

Komsta L. 2022. Moments, Cumulants, Skewness, Kurtosis and Related Tests. R Package. http://www.komsta.net/.

Lindroth CH (1949) Die Fennoskandischen Carabidae. Kungl. Vetensk. Vitterh. Samh. Handl. (Ser. B4) 1, Spezieller Teil. 1-709 (1945). 3, pp 1–911.

Löbl I, Löbl D (2018) Catalogue of Palaearctic Coleoptera. Volume 1: Archostemata – Myxophaga – Adephaga. Brill Academic Publishers, pp 1443. ISBN: 978-90-04-33029-0.

Müller-Motzfeld G (2004) Die Käfer Mitteleuropas, Bd. 2: Adephaga I: Carabidae. Springer Spektrum, pp 521. ISBN: 978-3-8274-1551-6.

Palmer AR 1994. Fluctuating Asymmetry Analyses: A Primer. In Developmental Instability: Its Origins and Evolutionary Implications, edited by T. A. Markow, pp. 335–364. Kluwer Academic Publishers.

Stanovský J, Pulpán J (2006) Střevlíkovití brouci Slezka (severovýchodní Moravy). [Invertebrate beetles of Silesia (northeastern Moravia).] Muzeum Beskyd Frýdek-Místek, pp 160.

Thiele HU (1977) Carabid Beetles in Their Environment. Springer-Verlag, Berlin, pp 369. https://doi.org.10.1007/978-3-642-81154-8.

Trautner J (2017). Die Laufäfer Baden-Württembergs. Verlag Eugen Ulmer, pp 848. ISBN: 978-3-8001-0380-5.

Turin H, Penev L, Casale A (eds.) (2003) The Genus Carabus in Europe. A Synthesis.- Co-published by Pensoft Publishers, Sofia-Moscow & European Invertebrate Survey, Leiden: xvi+ pp 512, 24 colour plates, 217 maps. The-genus-Carabus-in-Europe-A-Synthesis.pdf (researchgate.net).
